# Mechanistic insights into translation inhibition by aminoglycoside antibiotic arbekacin

**DOI:** 10.1093/nar/gkab495

**Published:** 2021-06-14

**Authors:** Narayan Prasad Parajuli, Chandra Sekhar Mandava, Michael Y Pavlov, Suparna Sanyal

**Affiliations:** Department of Cell and Molecular Biology, Biomedical Center, Uppsala University, SE-75124 Uppsala, Sweden; Department of Cell and Molecular Biology, Biomedical Center, Uppsala University, SE-75124 Uppsala, Sweden; Department of Cell and Molecular Biology, Biomedical Center, Uppsala University, SE-75124 Uppsala, Sweden; Department of Cell and Molecular Biology, Biomedical Center, Uppsala University, SE-75124 Uppsala, Sweden

## Abstract

How aminoglycoside antibiotics limit bacterial growth and viability is not clearly understood. Here we employ fast kinetics to reveal the molecular mechanism of action of a clinically used, new-generation, semisynthetic aminoglycoside Arbekacin (ABK), which is designed to avoid enzyme-mediated deactivation common to other aminoglycosides. Our results portray complete picture of ABK inhibition of bacterial translation with precise quantitative characterizations. We find that ABK inhibits different steps of translation in nanomolar to micromolar concentrations by imparting pleotropic effects. ABK binding stalls elongating ribosomes to a state, which is unfavorable for EF-G binding. This prolongs individual translocation step from ∼50 ms to at least 2 s; the mean time of translocation increases inversely with EF-G concentration. ABK also inhibits translation termination by obstructing RF1/RF2 binding to the ribosome. Furthermore, ABK decreases accuracy of mRNA decoding (UUC vs. CUC) by ∼80 000 fold, causing aberrant protein production. Importantly, translocation and termination events cannot be completely stopped even with high ABK concentration. Extrapolating our kinetic model of ABK action, we postulate that aminoglycosides impose bacteriostatic effect mainly by inhibiting translocation, while they become bactericidal in combination with decoding errors.

## INTRODUCTION

Inhibition of translation is one of the most common modes of action for a wide array of medically useful antibiotics. Many antibiotics inhibit protein synthesis in bacteria by binding in the vicinity of highly conserved functional centers of the bacterial ribosome (1,2). The aminoglycosides, a diverse set of ribosome-targeting antibiotics, are one of the most successful regimens in antimicrobial therapy of serious bacterial infections. Chemically, most of the aminoglycosides are natural or semisynthetic compounds derived from actinomycetes and share canonical 2-deoxystreptamine (DOS) core with an aminohexose sugar linked at 4-position (3,4). The minimal functional unit is deoxystreptamine, which directs aminoglycosides to bind near the decoding center of the ribosome, while the variety of appended moieties provide auxiliary contacts and regulate the strength and mode of their binding (5,6).

**Figure 1.**
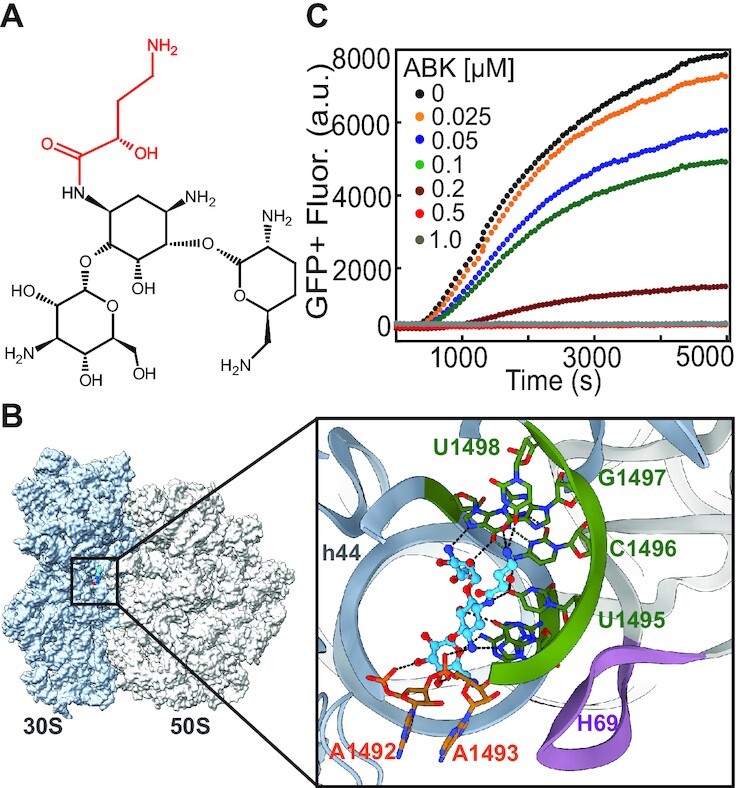
ABK chemical structure and the model of its binding to the bacterial ribosome. (**A**) Chemical structure of ABK with its amino-hydroxy butyrate (AHB) moiety in red. (**B**) Overview of the bacterial 70S ribosome with Amikacin (structurally similar to ABK) bound at helix 44 of 16S rRNA in the decoding center of 30S subunit. Enlarged image in the box is the detailed view of Amikacin (in blue) in its binding pocket. The conserved nucleobases A1492 and A1493 (orange) are flipped out from the helix 44. Additional interactions of the AHB moiety of the drug with 16S rRNA nucleobases U1495, U1496, G1497 and U1498 (*E. coli* numbering) can be seen. The figure is based on the cryo-EM maps of PDB IDs 6YPU and 6YHS. (**C**) Inhibition of in vitro GFP+ protein synthesis in an *E. coli* based reconstituted translation system induced by ABK added in different concentrations. Experiments were performed in duplicate and the averages are plotted.

Members of the classic (natural) aminoglycosides such as neomycin, kanamycin and paromomycin have constituted a major bulk of prescribed antibiotics in the clinics for years (6). However, despite the broad spectrum and excellent clinical efficacy, classic aminoglycosides nowadays have limited therapeutic use due to their adverse clinical effects and increasing global prevalence of resistance (7). Adverse effects are mainly due to their irreversible binding to the eukaryotic mitoribosome, as the drug-binding pocket of the mitoribosome differs, but little from their bacterial counterpart (8,9). In addition, the resistance to aminoglycoside arises through the action of several aminoglycoside modification enzymes (AMEs) carried on mobile elements, on plasmids or integrons (10,11) and due to the rRNA modifying enzymes that site-specifically methylate residues in rRNA to prevent the effective drug binding (12,13). In the effort to circumvent these issues, several first and second generations of semisynthetic aminoglycosides such as amikacin, arbekacin and plazomicin have been developed through chemical modification of classical aminoglycosides (6). Arbekacin (referred hereafter as ABK) (Figure 1A), a semisynthetic derivative of kanamycin B, developed as early as 1972 (14) and approved in Japan, Korea and USA for the treatment of multiple drug resistant (MDR) pneumonia and septicemia, is efficient against a wide range of bacterial pathogens (15,16). The (S)-4-amino-2-hydoxybutyryl (AHB) moiety of ABK attached at the N-1 DOS position, not only assures its stable binding to the ribosome (17,18) but also prevents the binding of several known aminoglycoside-modifying enzymes minimizing the risk of drug inactivation (14,15) and subsequent aminoglycoside resistance.

The mechanisms of aminoglycosides action on the ribosome and their global effects on protein synthesis in bacteria have been the subject of numerous studies in recent years (reviewed in (19,20)). It has been shown that many aminoglycosides bind to the conserved residues of helix 44 (h44) of 16S rRNA close to the decoding center (the A-site) of small ribosomal subunit (21–26) (Figure 1B). Their binding induces a conformational change in the decoding center, which greatly impairs the accuracy of decoding allowing promiscuous binding of near-cognate aminoacyl tRNAs (AA-tRNAs) (26–28) and hampers mRNA translocation (26,29–33). In addition, some aminoglycosides also bind to the major groove of helix 69 (H69) of 23S rRNA on the large ribosomal subunit (32,34,35). The latter binding leads to the distortion of inter-subunit bridge B2a that impairs the dynamics of subunit rotation and ribosome recycling (32,33,35). These investigations on classical and potentially toxic members of aminoglycoside have greatly enriched our understanding about their binding and the modes of inhibition of protein synthesis. However, there is dearth of comprehensive understanding about quantitative details of how aminoglycosides inhibit individual stages of the translation cycle. Likewise, relative impact of miscoding and inhibition of translocation on the global bactericidal effects of the aminoglycosides is less explored. Moreover, there is very little knowledge regarding mechanistic aspects of newer-generation therapeutic aminoglycosides such as ABK.

Here, we use fast kinetic methods to study the effects of ABK on elongation, termination and recycling steps of bacterial protein synthesis. We employed a cell-free translation system with *in vivo* like rate and accuracy reconstituted from translation components of high purity from *Escherichia coli*. Our results indicate that ABK induces errors in decoding and impedes ribosomal translocation with much higher efficacy than the previously characterized aminoglycosides (31–33). We have also found that ABK inhibits translation termination by interfering with the release factor (referred as RF) binding to the ribosome. Combining our experimental data, we suggest a quantitative kinetic model of ABK induced inhibition of protein synthesis on the bacterial ribosome. Our results also explain the bacteriostatic and bactericidal modes of action of aminoglycosides in general.

## MATERIALS AND METHODS

### Chemicals, buffers and translation components

All *in vitro* translation experiments were carried out at physiological temperature (37°C) in HEPES–Polymix (pH 7.5) buffer containing 5 mM HEPES (pH 7.5), 95 mM KCl, 5 mM NH_4_Cl, 5 mM Mg(OAc)_2_, 8 mM putrescine, 0.5 mM CaCl_2_, 1 mM spermidine and 1 mM 1,4-dithioerythritol. In addition, all the reaction mixtures were supplied with energy regeneration components such as 10 mM phosphoenol pyruvate (PEP), 50 μg/ml pyruvate kinase (PK), 2 μg/ml myokinase (MK) and variable amounts of ATP and GTP to ensure physiological range of the free Mg^2+^ concentration.

Tight-coupled 70S ribosomes of high purity and activity were prepared from JE28 *E. coli* cells using affinity-based purification (36). All in-house laboratory clones of translation factors (IF1, IF2, IF3, EF-Tu, EF-Ts, EF-G, RF1, RF2, RF3 and RRF) as well as Leucine and Phenylalanine aminoacyl tRNA synthetases were over-expressed in *E. coli* BL21 (DE3) cells and purified using Ni-IMAC affinity purification protocol (37). Concentrations of the ribosomes, translation factors, mRNA and tRNAs were measured spectrophotometrically. Initiator tRNA, f[^3^H]Met-tRNA^fMet^ and BODIPY^TM^ Met-tRNA^fMet^ were purified following the methods described in (37,38). Highly pure and active aminoacyl-tRNAs, tRNA^Leu^ and tRNA^Phe^ were prepared from overexpression of their in-house laboratory clone in MRE600 cells (37). The XR7-mRNAs with strong Shine-Dalgarno sequence (AAGGAGG) and a small ORF sequence **AUGUUCCUGUAA** (Met-Phe-Leu-stop), **AUGCUCUUCUAA** (Met-Leu-Phe-stop) and **AUGUUCUUCUUCUAA** (Met-Phe-Phe-Phe-stop) were prepared using *in vitro* transcription as in (39). Pyrene labelled mRNA (sequence 5′-UAACAAUAAGGGAGUAUUAA**AUGUUCCUG**C 3′-pyrene) coding for Met-Phe-Leu (40) were purchased from IBA-biosciences, Germany. ATP, UTP, CTP and GTP were from GE Healthcare. [^3^H]Methionine and [^3^H] GTP were from Perkin-Elmer. Arbekacin sulfate was from Carbosynth, United Kingdom-USP. Other chemicals were from Sigma-Aldrich or Merck.

### *In vitro* translation experiments

#### Reconstituted translation system for synthesis of a reporter protein GFP+

We prepared a cell-free reconstituted translation system (41) composed of active ribosomes (1 μM), translation factors (1–10 μM), amino acids, aminoacyl tRNA synthetases and tRNAs (bulk tRNA 100 μM) purified from *E. coli*. The reaction was started by the addition of mRNA encoding a reporter protein (GFP+). To test the effect of ABK on GFP+ synthesis, we added a range of ABK concentrations (0–1 μM) to the reaction mixture. The real time synthesis of GFP+ was monitored as the increase in fluorescence signal over time using a TECAN Infinite 200 PRO multimode plate reader.

#### Measurement of GTP hydrolysis on EF-Tu for cognate and near-cognate codons

Two separate reaction mixes were prepared. The initiation mix (IM) contained 70S ribosomes (0.4–2 μM), XR7-mRNA coding for Met-Phe-Leu-stop or Met-Leu-Phe-stop (UUC or CUC as second codon) (3 μM), initiation factors- IF1 (0.5–2 μM), IF2 (0.3–1 μM) and IF3 (0.5–2 μM), fMet-tRNA^fMet^ (0.5–2.5 μM), GTP (1 mM), and ATP (1 mM). The elongation mix (EM) contained EF-Tu (0.25–1.25 μM), tRNA^Phe^ (2.5 μM), Phe-RS (0.5 μM), phenylalanine (200 μM), [^3^H] GTP (0.25–1.25 μM) and ATP (2 mM). To study the ABK-induced effects, both IM and EM were supplied with various concentrations (0–40 μM) of ABK. Both mixes were incubated at 37°C for 15 minutes and equal volumes of each mix were rapidly mixed in a quench flow instrument (RQF-3 KinTek Corp.) followed by quenching the reaction with 17% formic acid (HCOOH) at different incubation intervals. Near-cognate reactions of GTP hydrolysis in the absence of ABK were slow, therefore mixing was carried out manually instead of quench flow. For precise estimation of kinetic parameters, a cognate reaction was always accompanied the near-cognate one. After quenching, samples were processed by centrifugation at 20 000 × g for 15 minutes at 4°C and the supernatants were used to estimate relative amounts of [^3^H] GTP and [^3^H] GDP. An anion-exchange chromatography Mono-Q GL column (GE Healthcare) connected to a Waters HPLC system coupled with in-line radioactive detector (LabLogic ß-RAM Model 4 IN/US) was used to analyze the samples. The mobile phase was a multistep gradient of 0–2 M NaCl in 20 mM Tris (pH 7.5) as described previously (39). The data were fitted with a single exponential function using Origin Pro 2016.

#### Measurement of cognate and near-cognate dipeptide formation

Similar to GTP hydrolysis, two mixes were prepared. The initiation mix (IM) contained 70S ribosomes (0.5 μM), XR7-mRNA Met-Phe-Leu-stop or Met-Leu-Phe-stop (UUC or CUC as second codon) (3 μM), IF1 (0.5 μM), IF2 (1 μM) and IF3 (0.5μM), f[^3^H]Met-tRNA^fMet^ (0.6 μM), GTP (1 mM) and ATP (1 mM). The elongation mix (EM) contained EF-Tu (2.5–12 μM), tRNA^Phe^ (0.5–10 μM), Phe-RS (0.5 μM), phenylalanine (200 μM), GTP (1 mM) and ATP (1 mM). For studying the effect of ABK, both IM and EM were supplied with various concentrations (0–40 μM) of ABK. Both mixes were incubated at 37°C for 15 minutes and equal volumes of each were rapidly mixed in a quench flow instrument (RQF-3 KinTek Corp.). The reactions were quenched with 17% HCOOH at different incubation times. For the much slower near-cognate reactions, mixing was carried out manually. All samples were centrifuged at 20 000 × g for 15 minutes in cold room (4°C). Supernatant was discarded and the peptides in the pellet were released from the tRNAs by adding 165 μl of 0.5 M KOH at room temperature. Cleaved tRNAs were precipitated (in ice) with 13.5 μl of 100% HCOOH and the samples were centrifuged again at 20 000 × g for another 15 min. The relative amounts of f[^3^H]Met and f[^3^H]Met-Phe in the supernatant were separated using a reverse phase chromatography column (C-18, Merck) connected to a Waters HPLC system coupled with in-line ß-RAM radioactive detector. The mobile phase for isocratic elution consisted of water, methanol and trifluoroacetic acid (58/42/0.1 v/v). The relative fractions of dipeptide were estimated by plotting the amount of fMet-Phe accumulation over time. The data were fitted with a single exponential function using Origin Pro 2016.

#### Measurement of dipeptidyl-tRNA drop-off from the A-site

The dropping off of the dipeptidyl-fMet-Phe-tRNA^Phe^ from the A-site was monitored by peptidyl-tRNA hydrolase (PTH) assay (42). The initiation mix (IM), containing 70S ribosomes (0.9 μM), f[^3^H] Met-tRNA^fMet^ (0.7 μM), XR7-mRNA (Met-Phe-Leu-stop) (3 μM), initiation factors (1 μM each), various concentrations of ABK (0–20 μM) and elongation mix (EM) of EF-Tu (5 μM), GTP (1 mM), Phe-tRNA^Phe^ (6 μM) and EF-G (0 or 5 μM) were prepared and incubated separately at 37°C for 15 minutes. PTH (15 μM) was then added to the EM and incubated for another 5 min. The reaction was started by mixing IM and EM and was quenched at different incubation times with 17% HCOOH. PTH hydrolyses peptidyl tRNA dropped off from the ribosome, thereby releasing peptides. The quenched samples were centrifuged at 20 000 × g for 15 min and the supernatant and pellet were separated. Pellets were processed similarly as for dipeptide experiments. Supernatants and pellets were subjected to RP-HPLC and relative fractions of radioactive peptides were estimated. Rate of peptidyl tRNA drop-off was estimated from the ratio of released peptide in the supernatant to the total peptide produced in the reaction. Data were fitted into single exponential function using Origin Pro 2016.

#### Measurement of EF-G catalyzed mRNA translocation

Initiation mix (IM) was prepared essentially in the similar way as in the case of dipeptide experiments, except that XR7-mRNA in IM was replaced with 3′ pyrene labeled mRNA (Met-Phe-Leu)+10 (43). The elongation mix (EM) contained EF-Tu (5 μM), EF-Ts (2 μM), EF-G (2.5- 80 μM), Phenylalanine (200 μM), Phe RS (0.5 μM), tRNA^Phe^ (12 μM), GTP (1 mM) and ATP (1 mM). ABK (0–10 μM) was added either to IM or EM as indicated. These two mixes were incubated at 37°C for 15 min. Equal volumes of IM and EM were rapidly mixed in a stopped-flow instrument (μSFM BioLogic) at 37°C and the fluorescence transition was monitored using 360-nm long-pass filter (Comar Optics Ltd.) after exciting at 343 nm as described earlier (44). The resultant fluorescence traces were fitted with a double exponential function using Origin Pro 2016.

#### Measurement of RF mediated peptide release

Pre-termination ribosome complex (Pre-TC) containing BODIPY™ (BOP)-Met-Phe-Leu-tRNA^Leu^ tripeptide in the P-site and a stop codon (UAA) in the A-site was prepared in HEPES polymix buffer (pH 7.5) (45). Briefly, initiation mix (IM) was prepared by incubating 70S ribosomes (2 μM), XR7-mRNA (Met-Phe-Leu-stop) (3 μM), BOP-Met-tRNA^fMet^ (2 μM), IF1 (2 μM), IF2 (4 μM) and IF3 (2 μM) at 37°C for 15 min. Similarly, elongation mix (EM) was prepared by incubating EF-Tu (20 μM), EF-Ts (20 μM), EF-G (10 μM), phenylalanine (200 μM), leucine (200 μM), aminoacyl tRNA synthetases (0.5 μM each), phenylalanine and leucine tRNAs (40 μM each) at 37°C for 15 min. Both mixes were supplied with energy regeneration components. Pre-TC was prepared by mixing IM and EM at 37°C for 5 min followed by quenching the reaction in ice. In order to stabilize the Pre-TC, additional Mg(OAc)_2_ (4 mM) was added to the mixture and the complex was purified by ultracentrifugation at 258 000 × g through a sucrose cushion (1.1 M) for 4 hours at 4°C. The pelleted pre-TC was resuspended in HEPES-Polymix buffer (pH 7.5) and stored at −80°C.

For peptide release experiments, equal volumes of pre-incubated pre-TC (0.1 μM) and RF mix containing RF1 or RF2 (1 μM) were rapidly mixed in a stopped-flow instrument (μSFM BioLogic) at 37°C. The release of BOP-Met-Phe-Leu tripeptide was followed by monitoring the decrease in BOP fluorescence (excitation: 575 nm) with a cutoff-filter of 590 nm (45). The fluorescence traces were fitted with double exponential function using Origin Pro 2016 and the rates were estimated from the predominant fast phase. To check the effect of ABK in peptide release, equal amount of ABK (0–10 μM) was added in both mixes. Similarly, to investigate the effect of ABK on RF binding to the ribosome, RF2 (1–20 μM) was titrated to pre-TC (0.1 μM) stalled with 10 μM ABK.

#### Measurements of ribosome recycling

Post-termination ribosome complex (post-TC) (with an empty A-site and deacylated tRNA in the P-site) was prepared by mixing 70S ribosomes (0.5 μM) with XR7- mRNA (Met-Phe-Leu-stop) (1 μM) and deacylated tRNA^Leu^ (1 μM) in HEPES–polymix buffer (pH 7.5). Similarly, factor mix (FM) was prepared by mixing RRF (40 μM), EF-G (20 μM) and IF3 (2 μM). Both mixes were incubated at 37°C for 5 min. Equal volumes of post-TC and FM were rapidly mixed in a stopped flow instrument (μSFM BioLogic) and splitting of post-TC into subunits was monitored as decrease in Rayleigh light scattering at 365 nm (46). The rate of post-TC dissociation was estimated by fitting the data with single exponential equation in Origin Pro 2016.

#### Measurements of ribosome turnover for tetrapeptide production

Two mixes were prepared. Initiation mix (IM) contained 70S ribosomes (0.1 μM), XR7-mRNA (Met-Phe-Phe-Phe-stop) (2 μM), initiation factors (0.5 μM each), f[^3^H]Met-tRNA^fMet^ (10 μM) and ABK (0- 1 μM). Factor mix (FM) contained EF-Tu (10 μM), EF-Ts (2 μM), EF-G (5 or 20 μM), Phe-tRNA^Phe^ (10 μM), RF2 (0.5 μM), RF3 (2 μM) and RRF (10 μM). Remaining components were as in the dipeptide experiments. The two mixes were incubated separately for 15 min at 37°C and the reaction was started by mixing equal volumes of each mix. At a definite time interval, an aliquot (40 μl) of the reaction mix was taken out and manually quenched with 17% HCOOH. The samples were processed and the released tetrapeptide (fMFFF) was analysed as described above for dipeptides. The tetrapeptide (picomoles) accumulated were plotted against time and fitted with single exponential function. The turnover time for ABK-free and ABK-stalled ribosomes was estimated by multiplying the linear slope of the initial phase of the reaction with the amount of active fraction of ribosomes (47).

## RESULTS

### ABK inhibits bacterial protein synthesis *in vitro*

We studied the effect of ABK on the process of mRNA translation by following the synthesis of a reporter protein GFP+ in an *E. coli* based reconstituted translation system (Materials and Methods). Increasing concentration of ABK reduced the amount of GFP+ produced with time (Figure 1C). ABK abolished GFP+ synthesis at sub-micromolar concentrations, inducing its half-maximal inhibitory effect (inhibitory constant, K_I_) at 125 nM. We have then extended our investigations to study the effect of ABK on all major steps of translation.

### ABK severely impairs the accuracy of AA-tRNA selection

**Figure 2.**
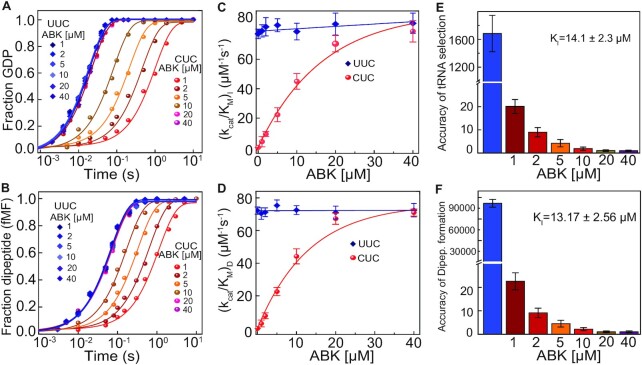
Effects of ABK on the fidelity of tRNA selection on the ribosome. (**A**) Time courses of GTP hydrolysis on EF-Tu ternary complex (*T*_3_) with [^3^H]GTP and Phe-tRNA^Phe^ during initial selection of tRNA on the 70S ribosomes programmed with cognate (UUC, bluish traces) and near-cognate (CUC, reddish traces) mRNA codons. ABK was present in the reaction as indicated. (**B**) Time courses of f[^3^H]Met-Phe dipeptide formation with ternary complex (*T*_3_) (1 μM) of EF-Tu, Phe-tRNA^Phe^ and GTP reacting to pre-initiated 70S ribosomes (0.5 μM) programmed with either cognate (UUC, bluish traces) or near-cognate (CUC, reddish traces) mRNA codon in their A-site. (**C**) Kinetic efficiencies (*k*_cat_/*K*_M_)_I_ of GTP hydrolysis on EF-Tu and (**D**) Kinetic efficiencies (*k*_cat_/*K*_M_)_D_ of dipeptide formation with cognate (blue) and near-cognate (red) reactions, estimated from experiments in A and B, respectively). (**E**) Accuracy of initial selection (I) of tRNA in the presence of ABK; estimated as the ratio between (*k*_cat_/*K*_M_)_I_ parameters for GTP hydrolysis with cognate and near-cognate codons. (**F**) Total accuracy (A) of the tRNA selection by the ribosome as estimated from the ratio of (*k*_cat_/*K*_M_)_D_ parameters for dipeptide formation with cognate and near-cognate ternary complexes. Solid lines in (**C**) and (**D**) are linear (blue) and hyperbolic (red) fits of *k*_cat_/*K*_M_ parameters for cognate and near-cognate reactions, respectively. Error bars represent the standard error of mean (SEM) values from at least three independent experiments.

To study the miscoding-inducing effects of ABK, we measured the kinetic efficiencies of (i) initial tRNA selection$({k_{{\rm cat}}}/{K_{\rm M}})_I^{}$, by monitoring GTP hydrolysis on EF-Tu and (ii) subsequent dipeptide formation $({k_{{\rm cat}}}/{K_{\rm M}})_{\rm D}^{}$ on the cognate as well as near-cognate codons. In these experiments, ternary complexes (*T*_3_) containing EF-Tu, GTP and Phe-tRNA^Phe^ were rapidly mixed in a quench flow instrument with the ‘initiation mix’ containing mRNA-programmed 70S ribosomes carrying fMet-tRNA^fMet^ in the P-site and a cognate (UUC) or near-cognate (CUC) codon in the A-site (Materials and Methods). The time courses of GTP hydrolysis on EF-Tu (Figure 2A) and dipeptide formation (Figure 2B) in the presence of various concentrations of ABK were used to estimate the kinetic efficiencies of the two reactions. The variations of measured cognate and near-cognate *k*_cat_/*K*_M_ parameters with increasing ABK concentration are shown in Figure 2C and Figure 2D for GTP hydrolysis and dipeptide formation, respectively. ABK, in the concentrations from 0 to 40 μM, had a negligible effect on the kinetic efficiency of GTP hydrolysis in case of the cognate codon (blue trace in Figure 2C), with $({k_{{\rm cat}}}/{K_{\rm M}})_I^c$ estimated as 79.2 ± 6.8 μM^–1^s^–1^, from the Y-axis intercept of the linear regression fit. In contrast, with the near-cognate codon, the $({k_{{\rm cat}}}/{K_{\rm M}})_I^{nc}$ for GTP hydrolysis increased dramatically with increase in ABK concentration from 0.072 ± 0.011 μM^–1^s^–1^ in the absence of ABK to a plateau value of 79.1 ± 7.29 μM^–1^s^–1^ at 40 μM ABK, matching the $({k_{{\rm cat}}}/{K_{\rm M}})_I^c$. The data could be fitted well with hyperbolic function (red trace in Figure 2C), and the concentration of ABK to reach the half-maximum (i.e. about 40 μM^–1^s^–1^) was 14.1 ± 2.3 μM. We interpret this value as the inhibitory constant (*K*_I_) of ABK at which half of the ribosomes were ABK-bound. The accuracy of initial tRNA selection, *I*, was estimated from the ratio of the kinetic efficiencies, $({k_{cat}}/{K_M})_I^c$/$({k_{{\rm cat}}}/K{}_{\rm M})_I^{nc}$. In absence of ABK, *I* was ∼1600 (Figure 2E), which matches well with a previous report (48). The accuracy dropped sharply to ∼20 upon addition of 1 μM ABK and then decreased gradually with further increase in ABK concentration (Figure 2E). The complete loss of accuracy of initial tRNA selection (i.e. *I* ∼ 1) was observed at about 40 μM ABK concentration.

Similar to the case of initial selection, ABK had a negligible effect also on the kinetic efficiency of dipeptide formation $({k_{{\rm cat}}}/{K_{\rm M}})_{\rm D}^c$ on cognate codon. The $({k_{{\rm cat}}}/{K_{\rm M}})_{\rm D}^c$ estimated from the Y-intercept of the linear fit (Figure 2D, blue trace) was 72.2 ± 2.2 μM^–1^ s^–1^. This value is virtually the same as $({k_{{\rm cat}}}/{K_{\rm M}})_I^c$ (Figure 2C), indicating negligible proofreading for cognate tRNA. The efficiency for near-cognate dipeptide formation, $({k_{{\rm cat}}}/{K_{\rm M}})_{\rm D}^{nc}$, increased hyperbolically with ABK concentration (Figure 2D, red trace), from a much lower value of 0.00086 ± 0.00011 μM^–1^ s^–1^ in the absence of ABK, to a saturating value of 71.0 ± 2.4 μM^–1^ s^–1^ at 40 μM of ABK, i.e. similar to $({k_{{\rm cat}}}/{K_{\rm M}})_{\rm D}^c$. The inhibitory constant (K_I_) estimated from the midpoint of hyperbolic fit was 13.17± 2.56 μM; the value also very similar to K_I_ for GTP hydrolysis (Figure 2C). From this, we calculate that the overall accuracy $A = ({k_{{\rm cat}}}/{K_{\rm M}})_D^c/({k_{{\rm cat}}}/{K_{\rm M}})_{\rm D}^{nc}$ dropped from 81 000 ± 5400 in the absence of ABK to 1.01 ± 0.042 at 40 μM ABK (Figure 2F), indicating that there was no discrimination against dipeptide formation with near-cognate AA-tRNA at this ABK concentration.

Importantly, in the presence of 1 μM and higher ABK concentrations, the accuracy of initial selection (Figure 2E) and the total accuracy (Figure 2F) were virtually identical. This implies that bound ABK stabilizes near-cognate AA-tRNAs in the decoding center of ribosome to such an extent that proofreading function of the decoding center is entirely lost (Figure 2E and F).

### ABK stabilizes dipeptidyl tRNA at the ribosomal A-site

The observation that ABK binding to the decoding center effectively abolishes its proofreading function (Figure 2E and F) could be explained by ABK-induced stabilization of codon-anticodon interactions in the A-site. Similar effect was also reported for a number of aminoglycosides (28,32) and tuberactinomycin (viomycin) (39). To corroborate this explanation, we studied the effect of ABK on the stability of dipeptidyl fMet-Phe-tRNA^Phe^ at the A-site of the ribosome. For this, we mixed EF-Tu•GTP•Phe-tRNA^Phe^ ternary complex with the ‘initiated’ ribosomes programmed with UUC (Phe) codon in the A-site and followed the dissociation of the dipeptidyl fMet-Phe-tRNA^Phe^ in the presence of peptidyl tRNA hydrolase (PTH), which hydrolyzes peptidyl-tRNA not bound to the ribosome (42). Our data (Supplementary Figure S1A) show that in the absence of ABK, fMet-Phe-tRNA^Phe^ dissociates from A-site with the rate of 0.45 ± 0.12 s^–1^ (dwell time of 2.2 s) (Supplementary Figure S1B). The mean dwell time increased to 16 s with 5 μM ABK, indicating that the stability of fMet-Phe-tRNA^Phe^ in the A-site increased by at least 8-fold. A further increase in ABK concentration up to 20 μM did not increase the mean dwell time of fMet-Phe-tRNA^Phe^ indicating that ribosomes have already been saturated with ABK at 5 μM.

### ABK inhibits EF-G catalyzed mRNA movement during ribosomal translocation

We next studied the effect of ABK on EF-G catalyzed mRNA movement during translocation using the fluorescent mRNA assay (43,44). For that, 70S ribosomes programmed with 3′-pyrene labeled mRNA coding for Met-Phe-Leu, and carrying fMet-tRNA^fMet^ in the P-site were rapidly mixed with EF-Tu•GTP•Phe-tRNA^Phe^ and EF-G in a stopped-flow instrument. The movement of the pyrene-labeled mRNA towards ribosomal mRNA tunnel results in fluorescence decrease, allowing one to monitor the time course of mRNA movement (43,44,49).

Fluorescent traces recorded in the absence of ABK and at different concentrations of EF-G in the reaction-mixture exhibited a near-monophasic fluorescence decay (Supplementary Figure S2A). From the rates of decay with 2.5, 5, 10 and 20 μM EF-G, estimated mean times for fMet-Phe-tRNA^Phe^ translocation were 99, 78, 64 and 56 milliseconds (ms), respectively (Supplementary Figure S2A). From these data we estimated *k*_cat_ = 22.8 ± 2.2 s^–1^ and *K*_M_ = 2.5 ± 0.3 μM (Supplementary Figure S2A, inset) for the translocation reaction in the absence of ABK. These values match closely the values of *k*_cat_ and *K*_M_ parameters of EF-G dependent translocation obtained by a different approach for other tRNAs and for codons both distant and close to the mRNA start codon (49). It implies that the rate measured in our assay reflects the rate of translocation irrespective of codon and its position in mRNA.

**Figure 3.**
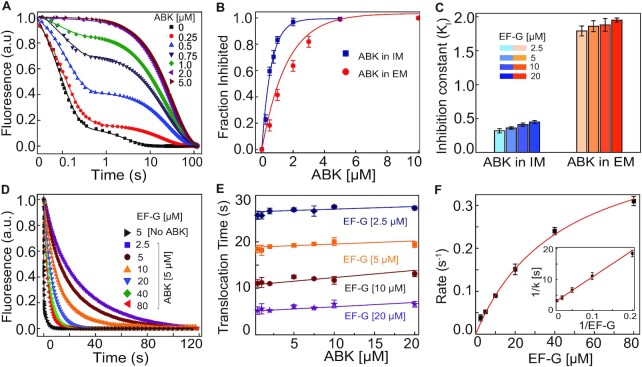
Effects of ABK on EF-G catalyzed mRNA translocation. (**A**) Real time fluorescence traces for the EF-G (5 μM) induced translocation of pyrene-labeled mRNA on 70S ribosome (0.5 μM). Ribosomes were pre-incubated with various ABK concentrations as indicated. The amplitudes and rates of fast and slow phases of fluorescence decrease were obtained from double exponential fit (solid lines) of experimental traces. The mean times of the fast and slow phases mRNA movement were estimated from the reciprocal of the rates. (**B**) The fraction of ABK inhibited ribosomes when ABK was added in the ‘initiation mix’ (IM, blue), and when it was added in the ‘elongation mix’ (EM, red). Solid lines represent hyperbolic fit of the data. (**C**) Inhibition constants (K_I_) for the ABK inhibition of translocation with various amounts of EF-G. (**D**) Time traces for pyrene-labeled mRNA translocation on the ABK (5 μM) saturated ribosomes with increasing concentrations of EF-G (2.5–80 μM). (**E**) Translocation times of pyrene-labeled mRNA on the ABK-stalled ribosomes at various ABK (0.5–20 μM) and EF-G (2.5–20 μM) concentrations, estimated from multiple experiments as in A. (**F**) Increase in the rates of mRNA translocation on the ABK-stalled ribosomes with increasing EF-G concentrations. The solid line is hyperbolic fit of the data. Inset shows the double reciprocal plot of the data for estimation of *k*_cat_ and *K*_M_ parameters. Error bars indicate the SEM of data obtained from at least three independent experiments.

When the ribosomes in the ‘initiation mix’ were pre-incubated with different concentrations of ABK, the time course of fluorescence decay became clearly biphasic. The rate of the fast phase matched closely the rate of fluorescence decay in the absence of ABK (Figure 3A). We therefore ascribed the fast phase of fluorescence decrease to the translocation on the ABK-free ribosomes, and the slow phase to the translocation on the ABK-bound ribosomes. Surprisingly, the rates of fast and slow phases did not vary with increasing ABK concentrations and corresponded to the translocation times of ∼80 ms in the absence, and ∼20 s (250-fold slower) in the presence of ABK, respectively (Figure 3A and Supplementary Figure S2B). All the fluorescence traces reached, however, the same final fluorescence level indicating the completion of mRNA translocation even in the presence of ABK. From the fluorescence amplitude of the slow phase, we estimated the fraction of the ABK-inhibited ribosomes at each ABK concentration (Figure 3B). This fraction increased hyperbolically with ABK concentration reaching half maximum (*K*_I_) at 0.36 ± 0.02 μM ABK. We concluded therefore that at this ABK concentration, half of the ribosomes were ABK-bound.

To estimate the speed of ABK binding to the ribosome in comparison with that of dipeptide formation and EF-G binding, we added ABK to the ‘elongation mix’, instead of pre-incubating it with the ‘initiation mix’. The fluorescence traces recorded in these experiments (Supplementary Figure S3) were essentially similar to those in Figure 3A, except that to reach the same extent of inhibition (the same fraction of inhibited ribosomes) a much higher ABK concentration was required. Accordingly, the hyperbolic increase of the inhibited fraction of ribosomes reached the half maximum (*K*_I_) at 1.83 ± 0.16 μM, 6-fold higher than when the ribosomes were pre-incubated with ABK (compare blue and red lines in Figure 3B). Interestingly, the inhibition constants (*K*_I_) did not vary with EF-G concentrations irrespective of whether ABK was added in the ‘initiation mix’ or ‘elongation mix’ (Figure 3C). It suggests that ABK binding occurs slower than EF-G binding to the pre-translocation ribosome.

### The translocation time on ABK-bound ribosomes depends on EF-G concentration

The time of mRNA movement during translocation on the ABK-inhibited ribosomes, estimated from the slow phase of the fluorescence traces, was ∼20 s at 5 μM EF-G concentration, irrespective of ABK concentration and whether it was added in the ‘initiation mix’ or ‘elongation mix’ (Figure 3A, Supplementary Figure S3). This surprising observation prompted us to study the behavior of the slow phase at varying concentration of EF-G (Figure 3D). The result of these ‘double titration’ experiments confirmed that at a fixed EF-G concentration, the mean time of translocation on the ABK-inhibited ribosomes remained indeed constant and did not vary with ABK concentration (Figure 3E). It varied, however, with EF-G concentration, decreasing from ∼26 s at 2.5 μM EF-G to ∼2 s at 80 μM EF-G (Figure 3D and E). The rate of mRNA translocation on the ABK-inhibited ribosomes increased hyperbolically with increasing EF-G concentrations to its maximum, *k*_cat_ = 0.48 ± 0.04 s^–1,^ reaching half of the maximum at *K*_M_ = 43 ± 5μM EF-G (Figure 3F). It indicates that pre-translocation ribosomes can remain simultaneously bound to ABK and EF-G for at least 2 s, after which ABK dissociation allows EF-G catalyzed translocation to proceed. Comparison of kinetic efficiencies, *k*_cat_*/K*_M_ of translocation reaction in the absence, *k*_cat_*/K*_M_ = 9.1 ± 1.6 μM^–1^s^–1^ (Supplementary Figure S2A, inset) and presence of ABK, *k*_cat_*/K*_M_ = 0.011 ± 0.008 μM^–1^ s^–1^ (Figure 3F) shows that ABK causes a more than 800 fold reduction in translocation efficiency.

### ABK impairs RF binding and inhibits peptide release

Since ABK binding distorts the decoding center of the ribosome, we thought to investigate its effect on peptide release with class-I release factors. To this end, pre-termination ribosome complexes (referred hereafter as pre-TC) containing BODIPY™ (BOP) labeled Met-Phe-Leu tripeptidyl tRNA in P-site and UAA stop codon in the A-site (Materials and methods) were purified and the time course of peptide release was followed by mixing them with RF1 or RF2 in a stopped-flow instrument. The exponential decrease in BOP fluorescence signifying single turn-over peptide release followed a nearly monophasic curve with a predominant (85–90% of the total amplitude) fast phase and a much slower slow phase (10–15% amplitude), that we ascribed to a small fraction of partially active ribosomes or mRNA in the reaction mixture.

**Figure 4.**
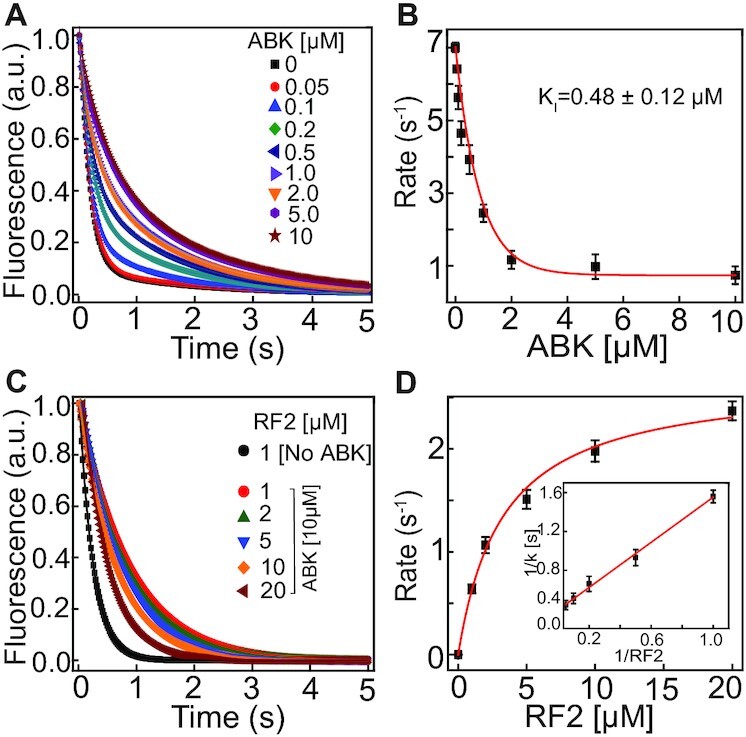
Effects of ABK on the release factor mediated peptide release. (**A**) BOP-fluorescence traces for the release of BOP-Met-Phe-Leu tripeptide from the ribosome by RF2 (1 μM) at indicated ABK concentrations. Solid lines are double exponential fits of the data. (**B**) Decrease in the rates of peptide release by RF2 (1 μM) with increasing concentrations of ABK. The rates are estimated from the predominant fast phase of the BOP-fluorescence traces. (**C**) BOP-fluorescence traces for the release of BOP-Met-Phe-Leu tripeptide from the ABK (10 μM) saturated ribosomes (0.1 μM) at indicated concentrations of RF2. (**D**) Hyperbolic plot showing increase in the rate of peptide release with increasing RF2 concentrations. The values of *k*_cat_ and *K*_M_ were estimated from the double reciprocal plot (inset). Error bars are SEM of the data obtained from at least two independent experiments.

Addition of ABK led to a near-hyperbolic decrease in the rate of fast phase with increasing ABK concentration (Figure 4A and Supplementary Figure S4A). The rate of peptide release approached a plateau at about 0.5 s^–1^ for RF1 and 1.0 s^–1^ for RF2 at ABK concentrations of ∼2 μM and remained virtually unchanged with further increase in ABK concentration. This implies that ABK, even at very high concentrations, could not completely block the RF-induced peptidyl tRNA hydrolysis on the ribosome. The inhibition constant *K*_I_, the concentration of ABK for half maximal inhibition, was 0.63 ± 0.23 μM for RF1 and 0.48 ± 0.12 μM for RF2 (Figure 4A and Supplementary Figure S4B).

We next studied the effect of variation in the RF2 concentration on the rate of peptide release in the presence of saturating ABK concentration (10 μM). In the absence of ABK, the rate of peptide release reached 7.4 ± 1.4 s^–1^ with 1 μM RF2 and remained virtually constant with increasing RF2 concentrations. In contrast, in the presence of 10 μM ABK the rate of peptide release was 0.3 s^–1^ at 1 μM RF2 and increased hyperbolically with RF2 concentration up to saturation at 2.6 ± 0.6 s^–1^, with half-maximal value reached at 3.2 ± 0.3 μM of RF2 (Figure 4B). From these plots, the *k*_cat_*/K*_M_ for peptide release with RF2 was estimated as 0.8 μM^–1^ s^–1^ at saturating ABK concentration, which was ∼30 fold smaller than *k*_cat_*/K*_M_ ≈ 20 ± 2.4 μM^–1^ s^–1^ in the absence of ABK. Taken together, these experiments suggest that ABK impedes RF binding to the pre-TC and thereby inhibits RF1 and RF2-induced peptide release. However, there is always a significant residual rate of peptide release even at saturating concentrations of ABK, which implies that a transient complex with the RF bound to ABK-containing ribosome can be formed at high RF concentrations. Occasionally, the ABK dissociation from this transient complex results in ABK-free RF-ribosome complex in which RF undergoes conformation change to its open, active state and positions its GGQ motif in the peptidyl transfer center (PTC) allowing peptide release (50,51).

### ABK inhibits post-termination ribosome recycling

The ribosome-recycling step occurs after peptide release, during which ‘ribosome recycling factor’ (RRF) together with EF-G split the post-termination ribosome complex (post-TC) into the subunits (52,53). To study the effect of ABK on the ribosome recycling step, the post-TC containing 70S ribosomes, mRNA and deacylated tRNA in the P-site was mixed rapidly with a reaction mix containing RRF, EF-G, and IF3 in a stopped-flow instrument. The time course of ribosome splitting was then monitored by following the decrease in Rayleigh light scattering (46) in the absence and presence of various concentrations of ABK (Supplementary Figure S5A). The traces were fitted with double exponential function and the ribosome splitting rates were estimated from the major fast phases. The rate of ribosome splitting decreased hyperbolically to zero with increase in concentration of ABK, decreasing to the half-maximal value at 30.4 ± 4.1 μM of ABK (Supplementary Figure S5B). These data show that ABK does inhibit ribosome recycling, albeit at much higher concentrations than other steps of the translation cycle.

### Effects of ABK on ribosome turnover for peptide production

**Figure 5.**
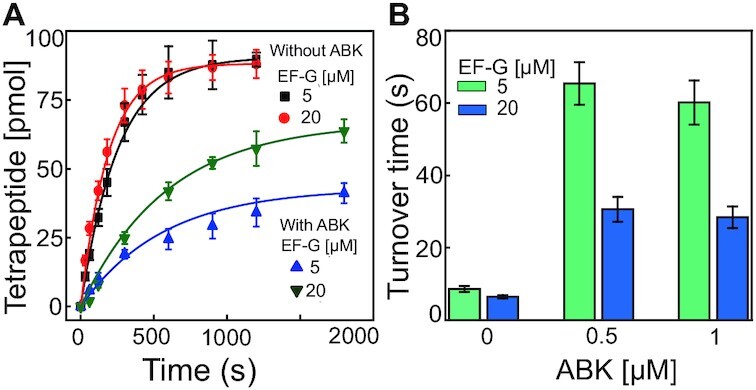
Effect of ABK on ribosome turnover for peptide production and its dependence on EF-G concentration. (**A**) Time evolution of the fMFFF tetrapeptide production with 70S ribosomes (0.1 μM) in multiple turnover conditions. Black and red traces are in the absence of ABK and blue and green traces in the presence of ABK (1 μM), respectively. The solid lines are single exponential fit of the data. (**B**) Times for ribosome turnover during tetrapeptide (fMFFF) production at indicated ABK and EF-G concentrations. Error bars represents the SEM of data obtained from at least two individual experiments.

Our kinetic experiments showed that ABK at saturating concentrations slows down mRNA translocation to 2 s or longer depending on EF-G concentration (Figure 3F), peptide release up to 400 ms (Figure 4D), and ribosome recycling up to 1 s (Supplementary Figure S5). Thus, translocation is a likely primary target for translation inhibition by ABK. To confirm this, we monitored the synthesis of a tetrapeptide (fMet-Phe-Phe-Phe) in a ribosome multi-turnover reaction in which all the components necessary for a complete cycle of peptide production were present (47) (Materials and Methods). The time course of fMFFF tetrapeptide accumulation was followed at two different concentrations of EF-G (5 and 20 μM), without and with ABK (0.5 and 1 μM) (Figure 5A). The ribosome turnover time was estimated from reciprocal of the rate of the tetrapeptide production. In absence of ABK, the turnover times were similar with 5 and 20 μM of EF-G, 8.6 s and 6.5 s, respectively. These times increased drastically to 65.5 s and 30.6 s, respectively, upon addition of 0.5 μM ABK (Figure 5B). Further increase in ABK concentration did not increase the turnover times (Figure 5B). Thus, addition of ABK prolonged the ribosome turnover times by 65.5 – 8.6 = 56.9 s in case of 5 μM EF-G, and 30.6 – 6.5 = 24.1 s for 20 μM EF-G. Interestingly, these times matched closely with the expected times for three ABK inhibited translocation events required to produce fMFFF peptide: 3×18 s = 54 s with 5 μM EF-G, and 3 × 6 s = 18 s with 20 μM EF-G (the values 18 s and 6 s were obtained in translocation experiments shown in Figure 3E). Thus, we conclude that the ABK induced delay in the peptide synthesis time is mainly due to the inhibition of mRNA translocation. This result also demonstrates that ABK inhibits overall protein synthesis even at a very low concentration. Considering that protein synthesis continues in the presence of ABK, albeit at a slow rate, we conclude that translocation inhibition alone cannot explain the bactericidal effects of aminoglycosides.

### Kinetic model for ABK induced inhibition of different steps of translation

**Figure 6.**
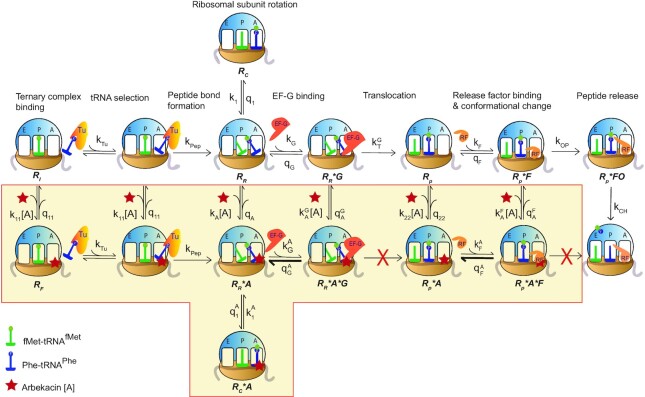
Kinetic model for ABK inhibition of different stages of translation. The *R_I_* and *R_F_* denote the ribosomal states where the monitoring bases A1492 and A1493 are in the ‘In-helix’ and ‘Flipped-out’ conformations, respectively. ABK binds to *R_I_* state with the rate constant *k_11_[A]* and induces its transition to *R_F_* state. The rate of dissociation of ABK from the *R_F_* state is *q_11_*. EF-Tu-ternary complex binds to *R_I_* and *R_F_* states with the rate constant *k_Tu_*, which leads to AA-tRNA selection and subsequent peptide bond formation. After peptide bond formation, ABK may stall the ribosomes in classical state denoted by R_C*_A, which hinders EF-G binding and translocation. However, at higher EF-G concentrations, a transient EF-G bound intermediate complex R_R*_A_*_G populates via R_R*_A state, where *R_R_* denotes the pre-translocation ribosome in rotated state. The inhibitory effect of ABK is enforced by preferential ‘backward’ dissociation of R_R*_A_*_G complex into R_R*_A and *G* with the rate $q_G^A$ (marked by a thick arrow) instead of its progression into R_R*_G state by the dissociation of ABK with the rate $q_A^G$. The R_R*_G state undergoes fast EF-G driven translocation with rate constant$k_T^G$. After completing the rounds of elongation, ABK binds to the pre-TC state *R_p_* and limit RF binding by forming R_P*_A complex. Similar to the case of translocation, higher RF concentrations induces the formation of the unstable R_P*_A_*_F complex that preferentially dissociates backwards to RF and R_P*_A with the rate $q_F^A$ (marked by a thick arrow). However, ABK will occasionally dissociate from R_P*_A_*_F with the rate $q_A^F$. The resulting R_P*_F complex with bound RF will undergo a fast conformational change with the rate *k_OP_* followed by peptide hydrolysis with the rate *k_CH_* (see supplementary text for details). The cross sign (in red) indicates the prohibited forward steps in translation cycle by ABK (box shaded in yellow).

Our results indicate that ABK can bind to ribosome essentially in all stages of the translation cycle. The experimental data led us to construct quantitative kinetic models to explain the mechanisms of ABK inhibition of tRNA selection, mRNA translocation and peptide release (Figure 6).

#### Effect on accuracy

ABK reduces the accuracy of tRNA selection in a concentration dependent manner (Figure 2). Our kinetic model predicts that ABK binding to 70S ribosome induces a hyperbolic increase in the near-cognate *k_cat_ / K_M_* parameters for GTP hydrolysis and dipeptide formation (*See* Scheme C in supplementary text). The ABK dependence of overall accuracy, *A*, can be approximated as $A = 1 + {K_I}/[ {ABK} ]$ for ABK concentrations above 0.1 μM (*see* supplementary text *for details)*. This means that the overall accuracy in the presence of ABK is simply determined by the inverse of the fraction of ABK-bound ribosomes in the reaction mixture (Figure 6).

#### Effect on translocation

Our results suggest that ABK binding to the pre-translocation ribosome hinders but does not completely abolishes EF-G binding (Figure 3 and Supplementary Figure S3). To model the effect of ABK on translocation, we assume that the ABK-bound ribosome in the rotated state (*R*_R_*A) (Figure 6) binds EF-G (denoted by G in Figure 6) slowly with the rate constant $k_G^A$ forming an unstable complex R_R_*A*G from which EF-G dissociates very fast with the rate constant $q_G^A$ so that the complex R_R_*A recovers. In contrast, ABK dissociates from the R_R_*A*G complex slowly with the rate constant $q_A^G$ which leads to ABK-free R_R_*G complex that undergoes fast translocation with the rate constant $k_T^G$_._ For such a model, the mean time of translocation, ${\tau _{TR}}$, is given by following expression (see supplementary text *for details*).

(1) $$\begin{eqnarray*}{\tau _{TR}} &=& \frac{1}{{k_T^G}} + \frac{{(1 + K_1^A)}}{{k_G^A\left[ G \right]}} + \frac{1}{{q_A^G}}\left\{ {1 + (1 + K_1^A)\frac{{K_G^A}}{{\left[ G \right]}}} \right\}\nonumber\\ &&\times\left(1 + \frac{{k_A^G\left[ A \right]}}{{k_T^G}}\right)\end{eqnarray*}$$

Here, $K_1^A = q_1^A/k_1^A$ is the equilibrium constant between rotated R_R_*A and classic R_C_*A states in the presence of ABK and $K_A^G = q_A^G/k_A^G$ is the equilibrium dissociation constant for EF-G binding to the rotated state of ABK-bound ribosome (with Peptidyl-tRNA in the A-site). In the derivation of Equation (1), we have also taken into account that right after dipeptide formation the ribosome is in the rotated state R_R_*A (see supplementary for details).

It follows from Equation (1) that the virtual constancy of the mean translocation time,${\tau _{TR}}$ at varying ABK concentration $[ A ]$ (Figure 3E) implies that at the ABK concentrations tested here, the rate, $k_A^G[ A ]$, of ABK rebinding to the pre-translocation complex R_R_*G is much slower than $k_T^G$. It also follows from Equation (1) that at increasing EF-G concentrations, the translocation time on ABK-bound ribosomes decreases and approaches its minimum, $1/k_T^G + 1/q_A^G$, where $1/q_A^G$ is the time of ABK dissociation from the unstable complex R_R_*A*G. Plotting the translocation time ${\tau _{TR}}$*versus* inverse of EF-G concentration, one obtains the value of 2 s from the Y-axis intercept (Figure 3F, inset). Further, since $k_T^G$ is faster than the rate of translocation in the absence of ABK, one can neglect the contribution of $1/k_T^G$ to the sum$1/k_T^G + 1/q_A^G$, which provides an estimate $q_A^G \approx$0.5 s^–1^ for the rate of ABK dissociation from (*R*_R_*A*G) complex.

#### Effect on termination

ABK affects termination by binding to the pre-TC (*R_p_*) with rate constant *k*_*A*_ forming R_p_*A complex that can revert to *R_p_* with rate constant *q_A_* upon ABK dissociation (Figure 6). During normal translation termination, release factor (RF) binds to pre-TC with rate constant *k_F_* forming R_p_*F complex (denoted by *F* is RF here and in the Figure 6), from which RF either dissociates with rate *q_F_* or changes to its open conformation with the rate constant *k*_OP_ and puts its GGQ motif in the PTC of the ribosome (Figure 6). This leads to the chemical reaction of ester bond hydrolysis between P-site tRNA and the peptide with the rate constant *k*_CH_ resulting in the release of peptide (54). In our model of termination in the presence of ABK, RF binds to ABK-bound pre-TC R_p_*A with the rate constant $k_A^F$ forming an unstable complex R_p_*A*F with ABK and RF. This complex preferentially dissociates back to R_p_*A with the rate $q_F^A$ or, occasionally, to R_p_*F with the rate $q_A^F$ (Figure 6). The R_p_*F complex can rebind ABK with the rate constant$k_A^F$$k_A^F$. This model leads to the following expression for the time of release ${\tau _{release}}$ (see supplementary text *for details*) at high ABK concentration (i.e., at ABK>2 μM):

(2) $$\begin{eqnarray*}{\tau _{release}} &=& \frac{1}{{{k_{OP}}}} + \frac{1}{{{k_{CH}}}} + \frac{1}{{k_F^A\left[ F \right]}} + \left(1 + \frac{{K_F^A}}{{\left[ F \right]}}\right)\frac{1}{{q_A^F}}\nonumber\\ &&\times\left(1 + \frac{{k_A^F\left[ A \right]}}{{{k_{OP}}}}\right)\end{eqnarray*}$$

Like in the case of translocation, Equation (2) shows that in the range of ABK concentrations where the rebinding rate of ABK, $k_A^F[ A ]$, to R_p_*F complex is much slower than the rate *k*_OP_ of RF2 ‘opening’ (*k*_OP_ ≈ 45 s^–1^ at 37°C (54)), ${\tau _{release}}$ will be insensitive to ABK concentrations. Moreover, it is observed from Equation (2) that the release time would decrease with increasing RF concentrations approaching the value:

${\tau _{release}} = 1/{k_{OP}} + 1/{k_{CH}} + 1/q_A^F$ (see supplementary text for details).

Thus, plotting the mean time of release *versu*s inverse of RF concentration, the minimal release time ${\tau _{release}}$, is estimated as 400 ms from the Y-intercept (Figure 4D, inset). Taking into account that the value 1/*k*_OP_ + 1/*k*_CH_ is estimated as 130 ms from the experiments in the absence of ABK (Figure 4A), we estimate the dissociation rate of ABK from R_p_*A*F complex containing ABK, RF and the ribosome as $q_A^F$= 3 s^–1^.

## DISCUSSION

Antibiotics inhibit bacterial translation through a variety of mechanisms. We clarified these mechanisms for the aminoglycoside antibiotic ABK by employing fast kinetic assays to monitor different stages of translation using a reconstituted bacterial translation system (39,40,55). Our results show that even at very high EF-G concentration ABK prolongs the mean time of translocation from ms range (without drug) to more than 2 s. This mean time increases well above 2 s at lower EF-G concentrations (Figure 3). This implies that in the presence of ABK, already at sub-micromolar concentrations, synthesis of a protein of average length of 300 amino acids, would take no less than 10 min, i.e. the time comparable with the doubling time of the fast-growing bacterial cells (56,57). Apart from inducing such a striking delay in ribosomal translocation, ABK also reduces the rate of peptide release at stop codons (Figure 4A), and impairs recycling of the post-terminated ribosomes (Supplementary Figure S5). However, since there is only one release and one recycling event, but multiple elongation events for synthesis of a full-length protein, the release/recycling damping effects of ABK seem to be negligible in comparison with its effects on elongation. Thus, impairment of bacterial translation by ABK is primarily due to the inhibition of translocation. Noteworthy, translocation (and also termination and recycling) do not stop completely even at the high ABK concentration but continue, albeit at a very slow rate. Thus, inhibition of translocation alone cannot account for the bactericidal property of ABK; rather it can explain its bacteriostatic properties.

Our results also demonstrate that ABK severely impairs the accuracy of mRNA translation. As evident from our data, already at 1 μM, ABK induces one miscoding error per 20 codons, in contrast to one miscoding per ∼80 000 codons in its absence (Figure 2F). Thus, ABK, at 1 μM concentration, would induce the incorporation of about 15 incorrect amino acids into a 300 amino acids long protein. Notably, the fidelity damping effect of ABK increases hyperbolically with ABK concentration (Figure 2C and 2D), reaching one incorrect amino acid per two correct ones at about 10 μM ABK. The obvious consequence of this would be synthesis of aberrant proteins with complete loss of function. This observation agrees well with a recent quantitative mass spectrometry analysis of cellular proteins, where aminoglycosides are shown to induce multitude of error clusters (58). Therefore, the ability of ABK to induce severe errors in decoding in combination with its dramatic effect on peptide elongation is the most likely cause of bacterial death.

Similar to other aminoglycosides, the effect of ABK on different steps of translation can be explained by the remodeling of the decoding center of the ribosome upon ABK binding (21,22,24,26). Essentially, the monitoring bases A1492 and A1493 flip-out of helix h44 of the 16S rRNA (21), which promotes 30S subunit domain closure (59) and concomitant GTP hydrolysis on EF-Tu, even when there is a mismatch between the codon and tRNA anticodon in the A-site (27,28). This leads to the acceptance of the near-cognate AA-tRNA and incorporation of erroneous amino acids in the synthesized polypeptide (60). Our observation, that the kinetic efficiencies of initial tRNA selection and dipeptide formation with near-cognate tRNAs are virtually identical for a given ABK concentration (Figure 2C and 2D), implies that the ribosomal proofreading is completely abolished in the presence of ABK. Our data also indicate that ABK stabilizes the peptidyl tRNAs in the A-site (Supplementary Figure S1). Combining these results, we conclude that ABK abolishes ribosomal proofreading by stabilizing the near-cognate tRNAs in the decoding center.

Similar to the loss of accuracy, inhibition of EF-G driven translocation can also be explained by ABK-induced flipping-out of A1492 and A1493, and stabilization of the peptidyl tRNAs. It is easy to envisage that strong stabilization of the tRNAs in the A-site will hinder their movement to the P-site, thereby inhibiting translocation (30,61). In addition, translocation requires disruption of the contacts between the codon-anticodon helix and the monitoring bases so that A1492 and A1493 can flip back inside helix h44 and open the gate for the codon- anticodon helix to move from the A- to the P-site (62–65). It has been shown that the docking of domain IV of EF-G near the decoding center promotes the disruption of these contacts allowing the monitoring bases to ‘flip in’ (66,67). This implies that ABK must dissociate from the decoding center to allow this ‘flipping in’ of the monitoring bases and the translocation to occur. It also implies that EF-G binding should promote ABK dissociation. Conversely, according to our kinetic model (Figure 6), ABK also disfavors EF-G binding by stabilizing the pre-translocation ribosome in the classical non-rotated state. Furthermore, EF-G and ABK can coexist on the rotated pre-translocation ribosome so that as soon as ABK dissociates, the already bound EF-G catalyzes translocation, which occurs with a speed much faster than ABK re-binding (Equation 1). This explains why increasing ABK concentrations do not cause higher inhibition of translocation at a given EF-G concentration (Figure 3E).

The effects of ABK on translocation differ markedly from those observed for another potent inhibitor of translocation, a tuberactinomycin - viomycin (Vio) (40,68,69). In contrast to ABK, translocation time increases with Vio concentration (40), which implies that Vio rebinds efficiently after its dissociation from pre-translocated ribosome. Moreover, the increase in EF-G concentration has no effect on translocation of the Vio-bound ribosomes, indicating that Vio binding does not interfere with EF-G binding (40). Thus, ABK and Vio inhibit translocation by different molecular mechanisms.

Interestingly, ABK induced inhibition of peptide release could be described by a kinetic model very similar to that of EF-G catalyzed translocation (compare Equation 1 and 2). In this model, ABK and RF can bind simultaneously to the pre-TC and form a transient complex. In this complex, the monitoring base A1493, flipped-out by ABK binding, clashes with the domain II of RFs preventing RFs from stop codon recognition and subsequent conformational change (opening) in RF required for peptide release (50,51). Occasionally, ABK dissociation from this complex allows stop codon recognition and peptide release by RFs. Again, ABK rebinding is potentially slower than conformational change in RF upon stop codon recognition, which explains the insensitivity of the peptide release rate to the increase in ABK concentration. By comparing the kinetic models for ABK inhibition of translocation and termination (Equations 1 and 2, Figure 6) we speculate that conformational change of the RFs upon codon recognition pushes back A1493 to the flipped-in conformation, which prevents ABK re-association. Structural validation of this model is a future perspective.

ABK can also inhibit the splitting of the ribosome into subunits at the recycling stage of translation cycle. This inhibition requires, however, higher-micromolar concentrations of ABK, which could be explained by the need of ABK binding to its low affinity secondary binding site near helix 69 (H69) of the large ribosomal subunit. This secondary binding is believed to stabilize the inter-subunit bridge (B2a/d) and impede the RRF induced displacement of H69, required for the inter-subunit bridge disruption and subunit separation (34).

In summary, our data, together with their kinetic analysis, provide clear evidence for ABK inhibition of various steps of translation cycle. Although ABK greatly reduces the kinetic efficiency of ribosomal translocation already at sub-micromolar concentrations, the translocation cannot be completely inhibited even at high concentrations of ABK. Thus, we conclude that translocation inhibition alone cannot explain the bactericidal effect of ABK and possibly of other aminoglycosides. However, in the higher range of ABK concentrations, the miscoding-inducing effect of ABK becomes so severe that it likely results in complete collapse of proteome. Since ABK binds to the canonical aminoglycoside-binding pocket at the decoding center of the ribosome and imparts monitoring base flipping in a generalized manner, our kinetic model for ABK inhibition can possibly be extrapolated to explain the mode of action of other aminoglycosides on bacterial translation. We propose, in accordance with recent *in vivo* studies (58,70), that drastic error induction in protein synthesis by aminoglycoside antibiotics in combination with severe translocation inhibition causes bacterial death. Our findings strengthen the efforts for rational development of aminoglycoside antibiotics and aid in further investigations of translational inhibitors in the era of looming antimicrobial resistance.

## DATA AVAILABILITY

All data are available in the main text or the supplementary materials. Additional data acquired during this study are available on request.

## Supplementary Material

gkab495_Supplemental_File
