# Supplementary material for: Mechanistic insights into translation inhibition by aminoglycoside antibiotic arbekacin
Source: Nucleic Acids Res. 2021 Jun 14;49(12):6880–92. doi: 10.1093/nar/gkab495 (PMC8266624; doi:10.1093/nar/gkab495)
Supplement: gkab495_Supplemental_File [file gkab495_supplemental_file.pdf]

SUPPLEMENTARY MATERIALS for

Mechanistic Insights into Translation Inhibition by Aminoglycoside Antibiotic Arbekacin

Narayan Prasad Parajuli, Chandra Sekhar Mandava, Michael Y. Pavlov, Suparna Sanyal*

Department of Cell and Molecular Biology, Biomedical Center, Uppsala University, SE-75124, Uppsala, Sweden

*To whom correspondence should be addressed: Tel: +46- 018471 4220. Fax: 018471 4262.
Email: suparna.sanyal@icm.uu.se

This PDF file includes:

Supplementary Text

1. Kinetic model for the ABK induced effect on tRNA selection, GTP hydrolysis and peptide bond formation
2. Kinetic model for ABK induced inhibition of EF-G catalyzed translocation
3. Kinetic model for ABK induced inhibition of RF mediated peptide release

Supplementary Figures

1. Supplementary Figure 1: Effect of ABK on stability of dipeptidyl-tRNA in the A-site of the ribosome.
2. Supplementary Figure 2: Kinetics of pyrene mRNA movement during EF-G catalyzed translocation.
3. Supplementary Figure 3: Effect of ABK on EF-G catalyzed mRNA translocation when ABK was added to "elongation mix".
4. Supplementary Figure 4: Effect of ABK on the release factor-1 (RF1) mediated peptide release.
5. Supplementary Figure 5: ABK induced inhibition of post-termination ribosome recycling.

Supplementary Text

1. Kinetic model for the arbekacin induced effect on the accuracy of tRNA selection on the ribosome

Kinetic schemes for GTP hydrolysis and peptide bond formation

The reaction of initial tRNA selection on the ribosome that leads to GTP hydrolysis on EF-Tu in the presence of ABK can be described by the following simplified kinetic scheme:

$$
\begin{array}{ccccccc}
R_I + T_3 & \underset{q_1}{\overset{k_1[T_3]}{\rightleftarrows}} & R_I * T_3^T & \underset{q_{20}}{\overset{k_{20}}{\rightleftarrows}} & R_I * T_3^C & \xrightarrow{k_{30}} & R_I * T_3^D \\
k_{11}[A]\downarrow\uparrow q_{11} & & k_{22}[A]\downarrow\uparrow q_{22} & & k_{33}[A]\downarrow\uparrow q_{33} & & \\
R_F + T_3 & \underset{q_1}{\overset{k_1[T_3]}{\rightleftarrows}} & R_F * T_3^T & \underset{q_2}{\overset{k_2}{\rightleftarrows}} & R_F * T_3^C & \xrightarrow{k_3} & R_F * T_3^D
\end{array}
$$

Scheme A. Kinetic model for the reaction of initial selection of tRNA on the ribosome

Here, the monitoring bases of the Ribosome (R) can be either in the "Flipped-out" or "In-helix" states ((1) and references therein) denoted here as R_F and R_I, respectively. ABK (A) binding to the R_I state with second order rate constant k_{11} flips out the monitoring bases resulting in the flipped-out state R_F. ABK dissociation from the R_F state with rate constant q_{11} returns the ribosome to the R_I state.

Without the drug, the ternary complex, T_3 , containing EF-Tu , GTP and AA-tRNA first binds with the second order rate constant k_1 into the ribosomal T-site (1) forming complexes denoted as $R_F * T_3^T$ or $R_I * T_3^T$ in Scheme A; $[T_3]$ here is the free concentration T_3 . There is no contact between the tRNA anti-codon and the A-site codon in either $R_F * T_3^T$ or $R_I * T_3^T$ ((1,2) and references therein) states, and, hence, the kinetics of the formation of these complexes is independent of whether the ribosome is in the R_F or R_I state. After initial binding, T_3 can dissociate from the initial-binding complex with the rate constant q_1. Alternatively, the AA-tRNA in the T_3 bends and its anti-codon forms the contact with the A-site codon in the decoding center of $R_I * T_3^T$ (1,2). This event results in the formation of the complexes $R_F * T_3^C$ and

$R_I * T_3^C$, where "C" means the codon-anticodon contact. The fate of these complexes is very different for the cognate and near-cognate tRNAs. In the case of cognate tRNA, the $R_I * T_3^C$ complex is converted into the $R_F * T_3^C$ complex regardless of the ABK presence, after which GTP hydrolysis on EF-Tu in T_3 occurs very fast with the rate k_3, resulting in the $R_F * T_3^D$ complex in which EF-Tu is bound to GDP (1,2). In contrast, in the case of near-cognate tRNA the codon-anticodon contact is unable to stabilize the flipped-out state of the monitoring bases so that the complex $R_I * T_3^C$ proceeds to GTP hydrolysis only very rarely , i.e. $k_{30} \approx 0$ (1,2). This near-cognate $R_I * T_3^C$ complex is also very unstable and, in most cases, reverts rapidly to the $R_I * T_3^T$ state with rate constant q_{20}; the instability implies that $q_{20} >> k_{20}$ and $q_{20} >> k_{33}[A]$. ABK binding to the $R_I * T_3^C$ complex converts it to the $R_F * T_3^C$ complex, which behaves similarly for both cognate and near-cognate tRNAs. Namely, since the monitoring bases have already been flipped out by ABK binding, $R_F * T_3^C$ will proceed rapidly to GTP hydrolysis irrespective of the nature (cognate or near cognate) of the tRNA in T_3 , meaning that $k_3 >> q_2$ and $k_3 >> q_{33}$. These considerations lead to the conclusion that for cognate tRNAs, both the top and bottom pathways from T_3 to GTP hydrolysis on EF-Tu (scheme A) will operate at similar kinetic efficiencies independent of ABK's presence, as also experimentally observed (Fig. 2). Conversely, in the case of near-cognate tRNAs the direct top reaction pathway involving $R_I * T_3^C$ formation is very inefficient so that the bottom reaction pathway (containing only R_F ribosomes and their complexes) will totally dominate the GTP hydrolysis kinetics in the presence of ABK. More precisely, under the conditions: $k_{30} \approx 0$, $q_{20} >> k_{20}$, $q_{20} >> k_{33}[A]$ and $k_3 >> q_{33}$ of the near-cognate case the state $R_I * T_3^C$ will not be populated in the presence of ABK and the kinetic scheme in figure A simplifies to:

$$
\begin{array}{ccccccc}
R_I + T_3 & \underset{q_1}{\overset{k_1[T_3]}{\rightleftarrows}} & R_I * T_3^T & & & & \\
k_{11}[A] \downarrow\uparrow q_{11} & & k_{22}[A] \downarrow\uparrow q_{22} & & & & \\
R_F + T_3 & \underset{q_1}{\overset{k_1[T_3]}{\rightleftarrows}} & R_F * T_3^T & \underset{q_2}{\overset{k_2}{\rightleftarrows}} & R_F * T_3^C & \xrightarrow{k_3} & R_F * T_3^D
\end{array}
$$

Scheme B. Simplified kinetic model for the reaction of GTP hydrolysis on near-cognate ternary complexes.

The scheme for dipeptide formation with near-cognate tRNA in the presence of ABK can also be viewed as a simple extension of scheme B for GTP hydrolysis.

$$
\begin{array}{ccccccccc}
R_I + T_3 & \underset{q_1}{\overset{k_1[T_3]}{\rightleftarrows}} & R_I * T_3^T & & & & R_F + AA-tRNA & & Dip \\
k_{11}[A] \downarrow\uparrow q_{11} & & k_{22}[A] \downarrow\uparrow q_{22} & & & & \uparrow q_p & & \uparrow k_D \\
R_F + T_3 & \underset{q_1}{\overset{k_1[T_3]}{\rightleftarrows}} & R_F * T_3^T & \underset{q_2}{\overset{k_2}{\rightleftarrows}} & R_F * T_3^C & \xrightarrow{k_3} & R_F * AA-tRNA & \xrightarrow{k_{acc}} & R_F * AA-tRNA_{acc}
\end{array}
$$

Scheme C. Simplified kinetic scheme for the reaction of dipeptide formation with near-cognate ternary complexes.

Here, the state $R_F * AA-tRNA$ with ribosome bound pre-accommodated AA-tRNA is equivalent to the state $R_F * T_3^D$ of the scheme B with T_3 in GDP form. This is because EF-Tu in GDP form releases the CCA end of AA-tRNA and dissociates from the ribosome allowing the AA-tRNA to either accommodate in the A-site of the large ribosomal subunit with the rate k_{acc} (state $R_F * AA-tRNA_{acc}$) and accept fMet from the P-site tRNA with the rate k_D or dissociate from the ribosome during the accommodation with rate q_P in the proofreading reaction (3,4).

A. Expressions for the mean time of dipeptide formation

We first calculate the mean time of dipeptide formation under our experimental condition when ternary complexes are in excess over ribosomes with free A-sites that are ready to accept T_3. In

this case, the kinetics of dipeptide formation in scheme C is governed by the follows set of differential equations:

$$\frac{d}{dt}c_{RI} = -(k_{11}[A] + k_1[T_3])c_{RI} + q_{11}c_{RF} + q_1 c_{RIT}$$
$$\frac{d}{dt}c_{RF} = k_{11}[A]c_{RI} - (q_{11} + k_1[T_3])c_{RF} + q_1 c_{RFT} + q_P c_{RD}$$
$$\frac{d}{dt}c_{RIT} = k_1[T_3]c_{RI} - (q_1 + k_{22}[A])c_{RIT} + q_{22}c_{RFT}$$
$$\frac{d}{dt}c_{RFT} = k_1[T_3]c_{RF} + k_{22}[A]c_{RIT} - (q_1 + q_{22} + k_2)c_{RFT} + q_2 c_{RFC}$$
$$\frac{d}{dt}c_{RFC} = k_2 c_{RFT} - (q_2 + k_3)c_{RFC} \qquad \text{(Eq. S1)}$$
$$\frac{d}{dt}c_{RD} = k_3 c_{RFC} - (k_{acc} + q_P)c_{RD}$$
$$\frac{d}{dt}c_{RAC} = k_{acc}c_{RD} - k_D c_{RAC}$$
$$\frac{d}{dt}c_{Dip} = k_D c_{RAC}$$

Here, we denoted as “c”, the ***deviations*** of concentrations of different complexes in the scheme C from their final concentration after the reaction completion. Those final concentrations are all zeros except for the concentration of the ribosomes containing dipeptides, which is equal to the initial concentration of the pre-initiated ribosomes. In the equation above we have also introduced the following notation for the free ribosomes and ribosome containing complexes: $RF = R_F$; $RI = R_I$; $RFT = R_F * T_3^T$; $RIT = R_I * T_3^T$; $RFC = R_F * T_3^C$; $RAC = R_F * AA - tRNA_{acc}$ and complex $RD = R_F * T_3^D = R_F * AA - tRNA$. The last notation is because the state $R_F * AA - tRNA$ with the ribosome bound pre-accommodated AA-tRNA is equivalent to the state $R_F * T_3^D$ of the scheme B with T_3 in the GDP form, as described above. Mean times τ_X are defined as in (2):

$$\tau_X = \frac{1}{C_{tot}} \int_0^{\infty} c_X(t) dt \qquad \text{(Eq. S2)}$$

Here, C_{tot} is total initial ribosome concentration. The concentration of free ternary complexes $[T_3]$ remains approximately constant during the reaction progress due to their large excess over the ribosomes so that we can integrate equation system of Eq. S1 from zero to infinite time considering $[T_3]$ constant. Taking into account the initial condition for the deviations of the concentrations, one obtains the following system of algebraic equations for the mean times:

$$\begin{aligned}
-\frac{K_{11}}{[A]+K_{11}} &= -(k_{11}[A]+k_1[T_3])\tau_{RI} + q_{11}\tau_{RF} + q_1\tau_{RIT} \\
-\frac{[A]}{[A]+K_{11}} &= k_{11}[A]\tau_{RI} - (q_{11}+k_1[T_3])\tau_{RF} + q_1\tau_{RFT} + q_P\tau_{RD} \\
0 &= k_1[T_3]\tau_{RI} - (q_1 + k_{22}[A])\tau_{RIT} + q_{22}\tau_{RFT} \\
0 &= k_1[T_3]\tau_{RF} + k_{22}[A]\tau_{RIT} - (q_1 + q_{22} + k_2)\tau_{RFT} + q_2\tau_{RFC} \\
0 &= k_2\tau_{RFT} - (q_2 + k_3)\tau_{RFC} \\
0 &= k_3\tau_{RFC} - (k_{acc} + q_P)\tau_{RD} \\
0 &= k_{acc}\tau_{RD} - k_D\tau_{RAC} \\
1 &= k_D\tau_{RAC}
\end{aligned} \qquad \text{(Eq. S3)}$$

Here K_{11} is the equilibrium dissociation constant for ABK binding to the ribosome:

$$K_{11} = q_{11} / k_{11} \qquad \text{(Eq. S4)}$$

We note the sum of deviation of the concentration is always zero due to the conservation law:

$$c_{RF}(t) + c_{RF}(t) + c_{RIT}(t) + c_{RFT}(t) + c_{RFC}(t) + c_{RD}(t) + c_{RAC}(t) + c_{dip}(t) = 0 \qquad \text{(Eq. S5)}$$

It follows from Eq. 5 that the mean time of dipeptide formation can be obtained as the sum of all other mean times:

$$\tau_{dip} = \tau_{RD} + \tau_{RF} + \tau_{RF} + \tau_{RIT} + \tau_{RFT} + \tau_{RFC} \qquad \text{(Eq. S6)}$$

Assuming that ABK binding does not depend on the initial binding of T_3 (since there in no disturbance of the decoding center by AA-tRNA of T_3 here), we may safely assume that $q_{22} = q_{11}$ and $k_{22} = k_{11}$ in scheme C. Solving the algebraic equation system Eq. S3 one obtains the following exact expression for the meantime, τ_{dip}, of dipeptide formation:

$$\frac{\tau_{dip}}{F} = \frac{1}{k_1[T_3]}\left[1 + \frac{q_1}{k_2}(1+\frac{q_2}{k_3})\right]\left(1+\frac{K_{11}}{[A]}\right) + \frac{1}{k_2}(1+\frac{q_2}{k_3})\left(1+\frac{K_{11}}{[A]}\right) + \frac{1}{k_3} + \frac{1}{k_{acc}F} + \frac{1}{k_D F}$$
$$+\frac{K_{11}}{[A]}\left[\frac{k_1[T_3]+q_1}{(k_{11}[A]+q_{11})\{q_{11}+k_{11}[A]+k_1[T_3]+q_1\}}\right] \quad \text{(Eq. S7)}$$
$$-\frac{q_{11}}{k_1[T_3]\{q_{11}+k_{11}[A]+k_1[T_3]+q_1\}}\left(1+\frac{K_{11}}{[A]}\right)$$

Here, $F = 1 + q_p / k_{acc}$ is the so-called proofreading factor ((3,4) and references therein). Neglecting the last two terms the sum of which is small in comparison with the first term in Eq. S7, one obtains the following approximation for the mean time of dipeptide formation:

$$\tau_{dip} \approx \frac{F}{k_1[T_3]}\left[1 + \frac{q_1}{k_2}(1+\frac{q_2}{k_3})\right]\left(1+\frac{K_{11}}{[A]}\right) + \frac{F}{k_2}(1+\frac{q_2}{k_3})\left(1+\frac{K_{11}}{[A]}\right) + \frac{F}{k_3} + \frac{1}{k_{acc}} + \frac{1}{k_D} \quad \text{(Eq. S8)}$$

At low $[T_3]$ concentration τ_{dip} is dominated by the first term in Eq. S8, so that in this $[T_3]$ concentration range the rate $k_{RG} = 1/\tau_{RG}$ of the accumulation of dipeptide in the reaction mixture is proportional to $[T_3]$:

$$k_{dip} \approx [T_3](k_{cat}/K_M)^{\max}_{dip} \cdot \left(\frac{1}{1+K_I/[A]}\right) \quad \text{(Eq. S9)}$$

Here, $K_I = K_{11}$ is an equilibrium dissociation constant of ABK binding to the ribosome and:

$$(k_{cat}/K_M)^{\max}_{dip} = \frac{1}{F}\frac{k_1}{[1+(q_1/k_2)(1+q_2/k_3)]} \quad \text{(Eq. S10)}$$

is the k_{cat} / K_M parameter of the bottom reaction pathway in the scheme C that only involves the ribosomal states with flipped-out monitoring bases and which is the major reaction pathway in the presence of ABK.

B. Expressions for the mean time of GTP hydrolysis

This case differs from the case of dipeptide formation in that the GTP hydrolysis experiments are conducted under conditions when the ribosomes are in a large excess over ternary complexes so that the rate of ribosome binding to ternary complexes is defined by free ribosome concentration and scheme B needs to be reformulated as

$$
\begin{array}{ccccccc}
R_I + T_3 & \underset{q_1}{\overset{k_1[R_I]}{\rightleftarrows}} & R_I * T_3^T & & & & \\
k_{11}[A] \downarrow\uparrow q_{11} & & k_{22}[A] \downarrow\uparrow q_{22} & & & & \\
R_F + T_3 & \underset{q_1}{\overset{k_1[R_F]}{\rightleftarrows}} & R_F * T_3^T & \underset{q_2}{\overset{k_2}{\rightleftarrows}} & R_F * T_3^C & \xrightarrow{k_3} & R_F * T_3^D
\end{array}
$$

Scheme D. Simplified kinetic model for the reaction of GTP hydrolysis on near-cognate ternary complexes for the case when ribosomes are in a large excess over ternary complexes.

Assuming also that the equilibrium between ABK bound and free ribosomes adjusts fast, the accumulation of $R_F * T_3^D$ complexes with GTP hydrolyzed to GDP is governed by the following system of differential equations:

$$
\begin{aligned}
\frac{d}{dt} c_{T3} &= -k_1 [R] c_{T3} + q_1 c_{RIT} + q_1 c_{RFT} \\
\frac{d}{dt} c_{RIT} &= (1-\alpha) k_1 [R] c_{T3} - (q_1 + k_{22}[A]) c_{RIT} + q_{22} c_{RFT} \\
\frac{d}{dt} c_{RFT} &= \alpha k_1 [R] c_{T3} + k_{22}[A] c_{RIT} - (q_1 + q_{22} + k_2) c_{RFT} + q_2 c_{RFC} \\
\frac{d}{dt} c_{RFC} &= k_2 c_{RFT} - (q_2 + k_3) c_{RFC} \\
\frac{d}{dt} c_{RD} &= k_3 c_{RFC}
\end{aligned}
\qquad \text{(Eq. S11)}
$$

Here, c_{T3} is the concentration of free T_3 and α is the fraction of ABK bound ribosomes that, being established during the pre-equilibration in the absence of T_3 , is maintained constant during the reaction progress:

$$\alpha = \frac{k_{11}[A]}{k_{11}[A]+q_{11}} \quad \text{(Eq. S12)}$$

Using that the ribosomes are in a considerable excess over ternary complexes T_3 we will neglect the reduction of $[R]$ as the reaction progresses and assume $[R]$ to be constant. We can then integrate the equation system Eq. S11 from zero to infinite time to obtain the system of algebraic equation for mean times. Solving this system for mean times and assuming, as before, that that $q_{22} = q_{11}$ and $k_{22} = k_{11}$ one obtains for the mean time of GTP hydrolysis which is the same as the meantime, τ_{RD}, of $R_F * T_3^D = RD$ complex accumulation:

$$\tau_{RD} = \frac{1}{k_1[R]}\left\{1+\frac{q_1}{k_2}(1+\frac{q_2}{k_3})\right\}\left(1+\frac{K_{11}}{[A]}\right)+\frac{1}{k_2}(1+\frac{q_2}{k_3})\left(1+\frac{K_{11}}{[A]}\right)+\frac{1}{k_3}+ \\ +\left(\frac{K_{11}}{[A]}\right)\frac{1}{(q_1+k_{11}[A]+q_{11})}-\frac{1}{k_1[R]}\left(1+\frac{K_{11}}{[A]}\right)\frac{q_{11}}{(q_1+k_{11}[A]+q_{11})} \quad \text{(Eq. S13)}$$

Neglecting the last two terms in comparison with the first one we get an approximation:

$$\tau_{RD} \approx \frac{1}{k_1[R]}\left\{1+\frac{q_1}{k_2}(1+\frac{q_2}{k_3})\right\}\left(1+\frac{K_{11}}{[A]}\right)+\frac{1}{k_2}(1+\frac{q_2}{k_3})\left(1+\frac{K_{11}}{[A]}\right)+\frac{1}{k_3}$$

In the range of ribosomal concentration where the first term dominates the rate $k_{RD} = 1/\tau_{RD}$ of the accumulation of GDP bound EF-Tu in the reaction mixture, is proportional to $[R]$:

$$k_{RD} \approx [R](k_{cat}/K_M)_{GTP}^{\max}\left(\frac{1}{1+K_I/[A]}\right) \quad \text{(Eq. S14)}$$

Here, $K_I = K_{11}$ is an equilibrium dissociation constant of ABK binding to the ribosome and:

$$\left(\frac{k_{cat}}{K_M}\right)_{GTP}^{\max} = \frac{k_1}{[1+(q_1/k_2)(1+q_2/k_3)]} \quad \text{(Eq. S15)}$$

Eq. S15 means that with increase in ABK concentration, the k_{cat} / K_M parameter of GTP hydrolysis reaction on near-cognate codon increases hyperbolically to its maximum value given by Eq. S15.

We note that Eq. S15 is analogues to Eq. S10 except that maximal k_{cat} / K_M of dipeptide formation is reduced by the proofreading factor F in comparison with the k_{cat} / K_M of GTP hydrolysis. Besides, the ribosome and not T_3 concentration defines the observed rate of GTP hydrolysis (compare Eq. 9 and 14).

We note also that the proofreading factor F also quantifies how many GTP hydrolysis events occur per peptide bond formation (3,4). Since near cognate AA-tRNA is stabilized in the A-site of the small subunit (q_P is small) by the presence of ABK and accommodates fast, the proofreading factor $F = 1 + q_p / k_{acc}$ is expected to be close to one (because $q_P << k_{acc}$).

2. Kinetic model for ABK induced inhibition of EF-G catalyzed translocation

We consider the translocation process in the presence of ABK using the following scheme;

$$
\begin{array}{ccccccc}
R_C & \underset{q_1}{\overset{k_1}{\rightleftarrows}} & R_R & \underset{q_G}{\overset{k_G[G]}{\rightleftarrows}} & R_R*G & \xrightarrow{k_T^G} & R_{TR} \\
k_A[A]\downarrow\uparrow q_A & & k_{11}[A]\downarrow\uparrow q_{11} & & k_A^G[A]\downarrow\uparrow q_A^G & & \\
R_C*A & \underset{q_1^A}{\overset{k_1^A}{\rightleftarrows}} & R_R*A & \underset{q_G^A}{\overset{k_G^A[G]}{\rightleftarrows}} & R_R*A*G & &
\end{array}
$$

Scheme E. Kinetic model for EF-G catalyzed translocation

Here, R_C is the ribosome with peptidyl-tRNA in the A-site in the classic state; R_R is the same ribosome in the rotated state; R_C*A and R_R*A are the ABK bound classic and rotated ribosomal states, respectively.

Our experimental data shows that ABK binding to the ribosome increases considerably the affinity of cognate peptidyl-tRNA to the A-site. From detailed balance consideration it then

follows that the presence of cognate peptidyl-tRNA in the A-site would increase ABK affinity to the decoding center, so that even at moderate ABK concentrations, ABK will be ribosome bound. This means that the ABK-free states R_C and R_R in the scheme above will not be populated and can be neglected. The scheme simplifies, therefore, to:

$$\begin{array}{ccccc} & & R_R*G & \xrightarrow{k_T^G} & R_{TR} \\ & & k_A^G[A]\downarrow\uparrow q_A^G & & \\ R_C*A & \underset{q_1^A}{\overset{k_1^A}{\rightleftarrows}} & R_R*A & \underset{q_G^A}{\overset{k_G^A[G]}{\rightleftarrows}} & R_R*A*G \end{array}$$

Scheme F. Simplified translocation scheme

Complex R_R*A binds EF-G slowly (since the binding is sterically hindered by presence of ABK) with the rate constant k_G^A forming an unstable state R_R*A*G. From this unstable state EF-G dissociates very fast with the rate constant q_G^A so that state R_R*A is recovered. The dissociation of ABK from the R_R*A*G state occurs much more slowly with the rate constant q_A^G and leads to state R_R*G with ABK-free ribosome and EF-G already bound. This R_R*G complex undergoes a very fast translocation with the rate constant k_T^G . The time evolution of the *Scheme F* is described by the following set of differential equations:

$$\begin{aligned}
\frac{d}{dt}c_{CA} &= -k_1^A c_{CA} + q_1^A c_{RA} \\
\frac{d}{dt}c_{RA} &= -(q_1^A + k_G^A[G])c_{RA} + k_1^A c_{CA} + q_G^A c_{RAG} \\
\frac{d}{dt}c_{RAG} &= k_G^A[G]c_{RA} + k_A^G[A]c_{RG} - (q_G^A + q_A^G)c_{RAG} \\
\frac{d}{dt}c_{RG} &= -k_A^G[A]c_{RG} + q_A^G c_{RAF} - k_T^G c_{RG} \\
\frac{d}{dt}c_{TR} &= k_T^G c_{RG}
\end{aligned} \qquad \text{(Eq. S16)}$$

Here, denoted as "c" the deviations of concentrations of different complexes from their final concentration after the reaction completion. Integrating these differential equations and taking

into account that initially all the ribosomes are in the state $R_R *A$ one obtains the following system of algebraic equations:

$$\begin{aligned}
0 &= -k_1^A \tau_{CA} + q_1^A \tau_{RA} \\
-1 &= -(q_1^A + k_G^A [G])\tau_{RA} + k_1^A \tau_{CA} + q_G^A \tau_{RAG} \\
0 &= k_G^A [G]\tau_{RA} + k_A^G [A]\tau_{RG} - (q_G^A + q_A^G)\tau_{RAG} \\
0 &= -k_A^G [A]\tau_{RG} + q_A^G \tau_{RAF} - k_T^G \tau_{RG}
\end{aligned} \qquad \text{(Eq. S17)}$$

After solving this system of algebraic equation to get all the mean times, the mean translocation time τ_{TR} is obtained as their sum:

$$\begin{aligned}
\tau_{TR} &= \tau_{CA} + \tau_{RA} + \tau_{RAG} + \tau_{RG} = \\
&= \frac{1}{k_T^G} + \frac{(1+K_1^A)}{k_G^A [G]} + \frac{1}{q_A^G}\left\{1 + (1+K_1^A)\frac{K_G^A}{[G]}\right\}(1 + \frac{k_A^G [A]}{k_T^G})
\end{aligned} \qquad \text{(Eq. S18)}$$

Here, $K_1^A = q_1^A / k_1^A$ is equilibrium constant between rotated and classic states in the presence of ABK and $K_G^A = q_G^A / k_G^A$ is the equilibrium dissociation constant for EF-G binding to the rotated state of ABK-bound ribosome (with Peptidyl-tRNA in the A-site).

Now, when ABK dissociates, the translocation in $R_R *G$ complex with already bound EF-G occurs very fast with the rate k_T^G so that ABK has virtually no chance to bind back with the rate constant k_A^G. This negligible ABK rebinding explains the insensitivity of translocation to ABK concentrations in micromolar range and the scheme effectively simplifies to:

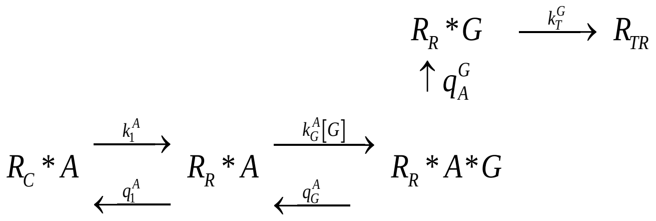

Scheme G. Simplified translocation scheme for the case of negligible ABK re-binding.

The rate limiting step here is the ABK dissociation from the unstable R_R*A*G complex with the rate constant q_A^G =0.5 s^{-1} (*Scheme G*) meaning that ABK dwells for the minimum of 2 s in the unstable R_R*A*G state.

3. Kinetic model for ABK induced inhibition of RF mediated peptide release

We used the following notation for scheme of RF mediated peptide release:

$$\begin{array}{ccccc}
R_p & \underset{q_F}{\overset{k_F[F]}{\rightleftarrows}} & R_p*F & \xrightarrow{k_{OP}} & R_p*FO \\
k_{22}[A]\downarrow\uparrow q_{22} & & k_A^F[A]\downarrow\uparrow q_A^F & & \downarrow k_{CH} \\
R_p*A & \underset{q_F^A}{\overset{k_F^A[F]}{\rightleftarrows}} & R_p*A*F & & Pept
\end{array}$$

Scheme H: Kinetic model for RF mediated peptide release

Here, pre-termination ribosome R_p can bind ABK (A) with the second rate constant k_{22} forming R_p*A complex that can revert to R_p with rate constant q_{22} upon ABK dissociation. Release factor (F) binds to pre-termination ribosome with rate constant k_F forming R_p*F complex, from which F either dissociates with rate q_F or changes its conformation ("opens") with the rate constant k_{OP} and puts its GGQ motive in the peptidyl transferase center of the ribosome. This leads to the chemical reaction of ester bond hydrolysis between P-site tRNA and peptide with rate constant k_{CH} leading to the peptide release (1,5). Similarly, ABK containing pre-termination ribosome (R_p*A) can also bind F with rate constant k_F^A forming an unstable complex R_p*A*F with ABK and release factor. This complex dissociates preferentially back to R_p*A with rate q_F^A or occasionally dissociate to R_p*F with rate q_A^F. The R_p*F complex can rebind ABK with rate constant k_A^F.

The kinetics of Scheme H is described by the following set of differential equations:

$$\begin{aligned}
\frac{d}{dt}c_R &= -(k_A^F[A]+k_F[F])c_R + q_A^F c_{RA} + q_F c_{RF} \\
\frac{d}{dt}c_{RA} &= k_A^F[A]c_R - (q_A^F + k_F^A[F])c_{RA} + q_F^A c_{RAF} \\
\frac{d}{dt}c_{RF} &= k_F[F]c_R - (q_F + k_A^F[A] + k_{OP})c_{RF} + q_A^F c_{RAF} \\
\frac{d}{dt}c_{RAF} &= k_F^A[F]c_{RA} + k_A^F[A]c_{RF} - (q_F^A + q_A^F)c_{RAF} \\
\frac{d}{dt}c_{RFO} &= k_{OP}c_{RF} - k_{CH}c_{RFO} \\
\frac{d}{dt}c_P &= k_{CH}c_{RFO}
\end{aligned} \qquad \text{(Eq. S19)}$$

Here, the subscripts are: $RA=R_p*A$; $RF=R_p*F$; $RAF=R_p*A*F$; $RFO=R_p*FO$. Since both ABK and RF (F) are present in a large excess over pre-termination complexes with free A-sites (ready to accept RF) we can consider their concentrations constant and integrate the differential equation system as in Eq. S2. One obtains the following algebraic equation system for mean times:

$$\begin{aligned}
-\alpha &= -(k_A^F[A]+k_F[F])\tau_R + q_A^F\tau_{RA} + q_F\tau_{RF} \\
\alpha - 1 &= k_A^F[A]\tau_R - (q_A^F + k_F^A[F])\tau_{RA} + q_F^A\tau_{RAF} \\
0 &= k_F[F]\tau_R - (q_F + k_A^F[A] + k_{OP})\tau_{RF} + q_A^F\tau_{RAF} \\
0 &= k_F^A[F]\tau_{RA} + k_A^F[A]\tau_{RF} - (q_F^A + q_A^F)\tau_{RAF} \\
0 &= k_{OP}\tau_{RF} - k_{CH}\tau_{RFO} \\
1 &= k_{CH}\tau_{RFO}
\end{aligned} \qquad \text{(Eq. S20)}$$

Here, "α" is the initial fraction of ABK free post-termination ribosomes before RF addition. Solving this system of algebraic equation to get all mean times, the mean release time is then obtained as their sum:

$$\tau_{release} = \tau_R + \tau_{RA} + \tau_{RAF} + \tau_{RF} + \tau_{RF0} =$$
$$= \frac{1}{k_F[F]}(1-\beta) + \beta\left(\frac{1}{k_F^A[F]} + \frac{1}{k_F^A[F]}\frac{q_F^A}{q_A^F} + \frac{1}{q_A^F}\right) + \qquad \text{(Eq. S21)}$$
$$+ \frac{1}{k_{OP}} + \frac{1}{k_{CH}} + \frac{1}{k_F[F]}\frac{q_F}{k_{OP}} + \frac{1}{q_A^F}\left(1+\frac{q_F^A}{k_F^A[F]}\right)\frac{k_A^F[A]}{k_{OP}}$$

Here:

$$\beta = \left(\frac{k_{22}[A]}{k_{22}[A]+q_{22}} + \frac{k_{22}[A]}{k_F[F]}\right) / \left\{1 + \frac{k_{22}[A]}{k_F[F]} + \frac{q_A^F}{k_F[F]}\left(1+\frac{q_F^A}{q_A^F}\right)\right\}$$

In the case when the pre-terminated ribosomes are saturated with ABK, β is very close to 1 and the expression for the mean release time simplifies to:

$$\tau_{release} = \frac{1}{k_F^A[F]} + \frac{1}{q_A^F}\left(1+\frac{q_F^A}{k_F^A[F]}\right)\left(1+\frac{k_A^F[A]}{k_{OP}}\right) + \frac{1}{k_{OP}}\left(1+\frac{q_F}{k_F[F]}\right) + \frac{1}{k_{CH}} \qquad \text{(Eq. S22)}$$

We further note that when the RF concentration is high enough to saturate the ABK-free reaction (i.e. when $q_F / k_F << [F]$), the above equation simplifies further to:

$$\tau_{release} = \frac{1}{k_{OP}} + \frac{1}{k_{CH}} + \frac{1}{k_F^A[F]} + \frac{1}{q_A^F}\left(1+\frac{K_F^A}{[F]}\right)\left(1+\frac{k_A^F[A]}{k_{OP}}\right) \qquad \text{(Eq. S23)}$$

Here, $K_F^A = q_F^A / k_F^A$ is the equilibrium-binding constant of RF to the ABK containing ribosome.

Taking also in account that in our experiments $k_A^F[A] << k_{OP}$ one gets:

$$\tau_{release} = \frac{1}{k_{OP}} + \frac{1}{k_{CH}} + \frac{1}{k_F^A[F]} + \frac{1}{q_A^F}\left(1+\frac{K_F^A}{[F]}\right) \qquad \text{(Eq. S24)}$$

Supplementary Figures

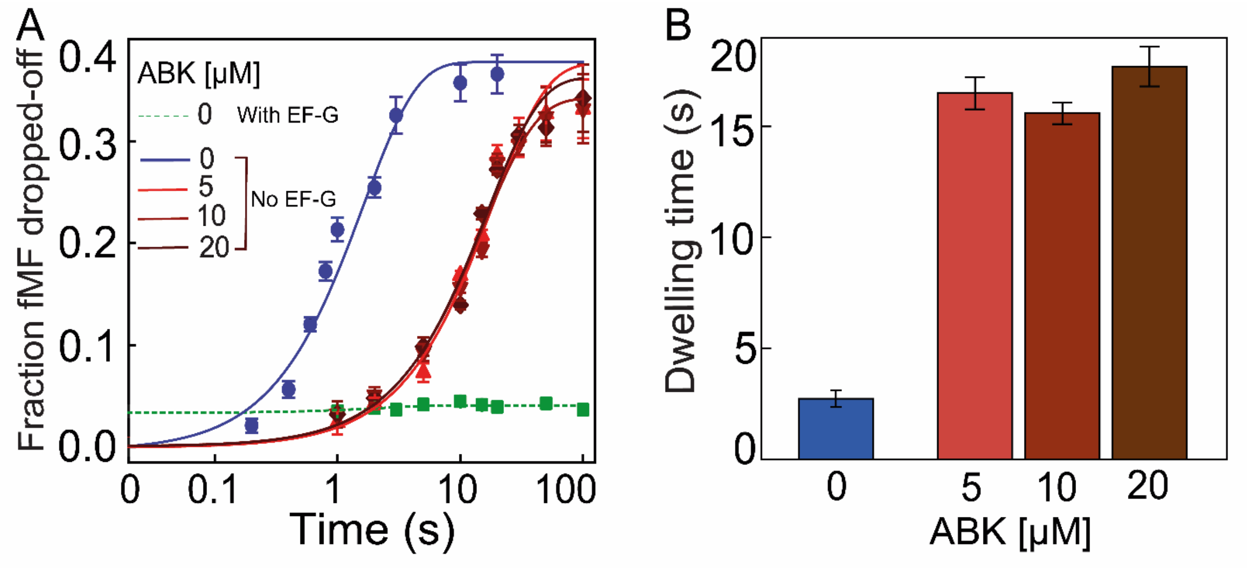

Supplementary Figure 1. Effect of ABK on stability of dipeptidyl-tRNA in the A-site of the ribosome.

(**A**) Time courses for dipeptidyl fMet-Phe-tRNA drop-off from the A-site of the ribosome. The reaction was started by adding EF-Tu•GTP•Phe-tRNA^{Phe} ternary complex (5 µM) together with peptidyl tRNA hydrolase (PTH) (10 µM) to an initiation mix containing 70S ribosome (0.7 µM) programmed with XR7-mRNA coding for Met-Phe-Leu-STOP, fMet-tRNA^{fMet} in the P-site and the indicated concentrations of ABK. As a positive control, the reaction was conducted in the presence of EF-G (5 µM), to translocate the dipeptidyl fMet-Phe –tRNA^{Phe} into the P-site from where only an extremely slow drop-off occurs. The solid lines represent exponential fit of the data and error bars are SEM.

(**B**) Dwell times of the dipeptidyl-tRNA (fMet-Phe-tRNA^{Phe}) at the A-site of ribosome at different concentrations of ABK. Error bars are SEM of the data.

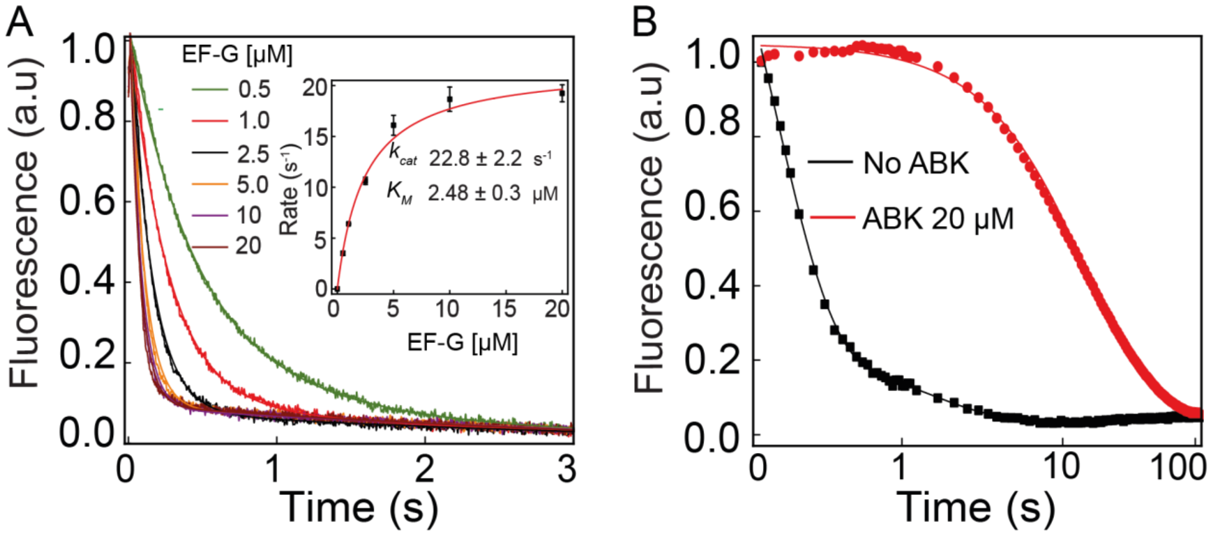

Supplementary Figure 2. Kinetics of pyrene mRNA movement during EF-G catalyzed translocation.

(**A**) Fluorescence time traces for pyrene mRNA movement on pre-translocation ribosomes (0.5 µM) in the absence of ABK with increasing concentrations of EF-G (0.5- 20 µM). The data were fitted with double exponential function and the rates were estimated from the predominant fast phase. The rates increased hyperbolically with EF-G concentration (inset) allowing determination of the k_{cat} =22.8 ± 2.2 s^{-1} and K_M =2.48 ± 0.3 µM parameters. The mean-times of mRNA movement at a particular EF-G concentration were obtained from the reciprocal of the rates. (**B**) Effect of ABK (20 µM) on EF-G (5 µM) catalyzed translocation of pyrene mRNA on the ribosome (0.5 µM). Solid lines are double exponential fit of the data.

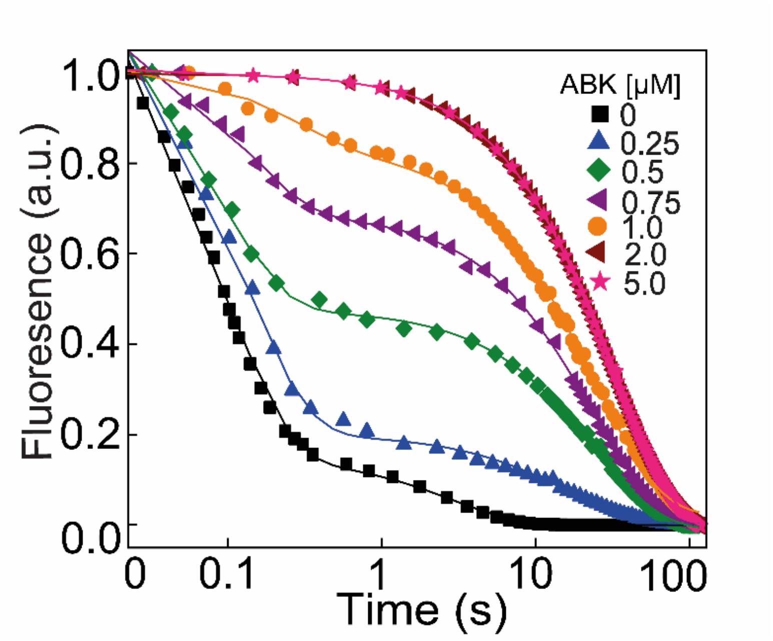

Supplementary Figure 3. Effect of ABK on EF-G catalyzed mRNA translocation when ABK was added to "elongation mix". Real time fluorescence traces for the EF-G (5 μM) catalyzed translocation of pyrene-mRNA on 70S ribosome (0.5 μM). Indicated concentrations of ABK were present only in the elongation mix while ribosomes were free of ABK prior to mixing. The amplitudes and rates of fast and slow phases of fluorescence decrease were obtained from double exponential fit (solid lines) of experimental traces. The mean times of the fast and slow phases mRNA movement were estimated from the reciprocal of the rates.

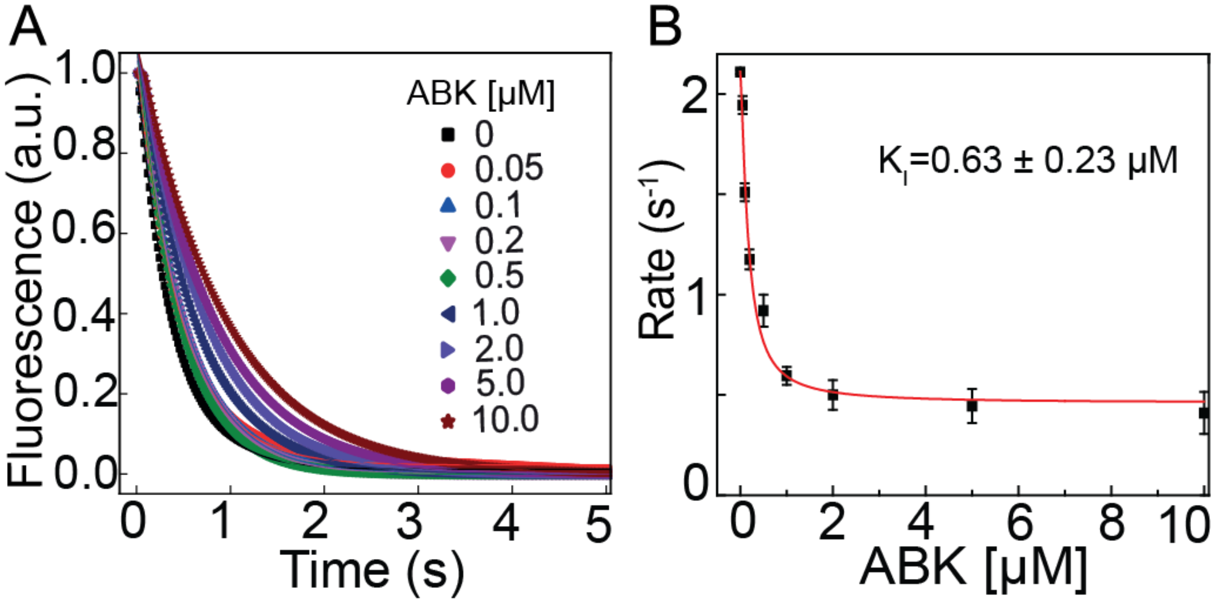

Supplementary Figure 4. Effect of ABK on the release factor-1 (RF1) mediated peptide release. (**A**) BOP-fluorescence traces for the release of BOP-Met-Phe-Leu tripeptide from the ribosome by RF1(1 µM) at indicated ABK concentrations. Solid lines are double exponential fits of the data. (**B**) Decrease in the rates of peptide release by RF1 (1 µM) with increasing concentrations of ABK. The rates of peptide release were estimated from the predominant fast phase of the BOP-fluorescence traces in **(A)**. Error bars are SEM of data.

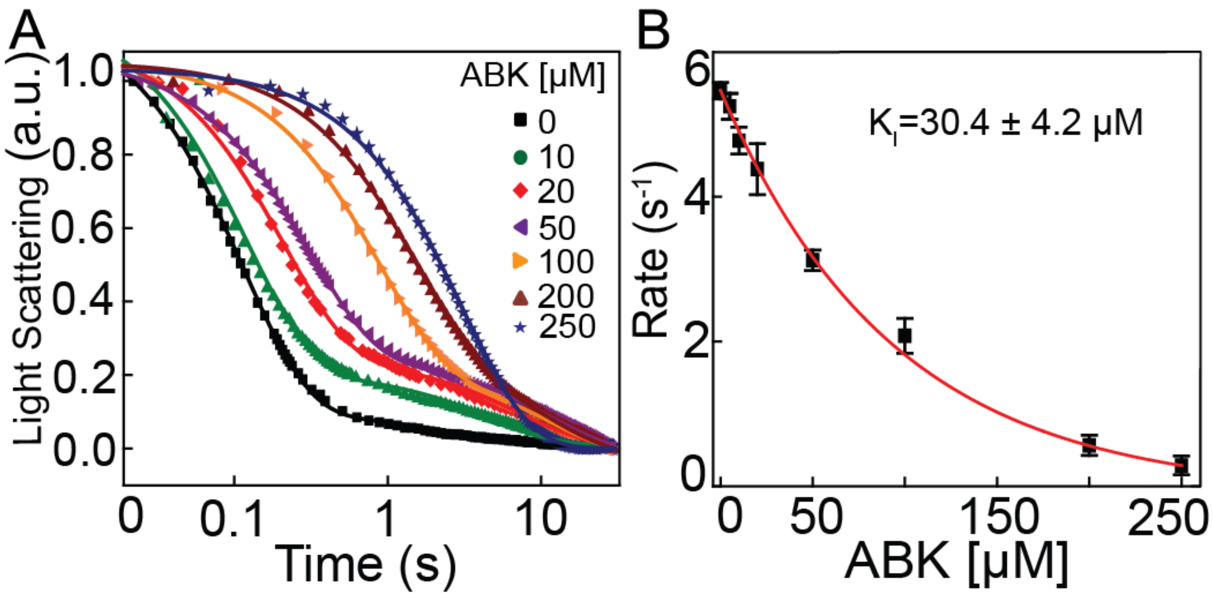

Supplementary Figure 5. ABK induced inhibition of post-termination ribosome recycling. **(A)** Time traces of splitting of post-termination 70S ribosome (0.5 μM) into subunits by ribosome recycling factor RRF (20 μM) and EF-G (10 μM) at indicated ABK concentrations. Solid lines are double exponential fit of data. The predominant fast phase was used to estimate the rates of ribosome recycling at various ABK concentrations. **(B)** Decrease in rate of ribosome recycling with increasing concentration of ABK fitted with a hyperbolic function. The inhibition constant (K_I) is the concentration of ABK required to reduce the rate of ribosome recycling to its half. Error bars are SEM of the data.

REFERENCES

1. Pavlov, M.Y. and Ehrenberg, M. (2018) Substrate-Induced Formation of Ribosomal Decoding Center for Accurate and Rapid Genetic Code Translation. *Annu. Rev. Biophys.*, **47**, 525-548.
2. Zhang, J., Pavlov, M.Y. and Ehrenberg, M. (2018) Accuracy of genetic code translation and its orthogonal corruption by aminoglycosides and Mg2+ ions. *Nucleic Acids Res.*, **46**, 1362-1374.
3. Zhang, J., Ieong, K.W., Mellenius, H. and Ehrenberg, M. (2016) Proofreading neutralizes potential error hotspots in genetic code translation by transfer RNAs. *RNA* **22**, 896-904.
4. Pavlov, M.Y., Liljas, A. and Ehrenberg, M. (2017) A recent intermezzo at the Ribosome Club. *Philos. Trans. R. Soc. Lond. B. Biol. Sci.*, **372**.
5. Indrisiunaite, G., Pavlov, M.Y., Heurgué-Hamard, V. and Ehrenberg, M. (2015) On the pH dependence of class-1 RF-dependent termination of mRNA translation. *J. Mol. Biol.*, **427**, 1848-1860.
